# Functional Requirements for Heparan Sulfate Biosynthesis in Morphogenesis and Nervous System Development in *C*. *elegans*

**DOI:** 10.1371/journal.pgen.1006525

**Published:** 2017-01-09

**Authors:** Cassandra R. Blanchette, Andrea Thackeray, Paola N. Perrat, Siegfried Hekimi, Claire Y. Bénard

**Affiliations:** 1 Department of Neurobiology, UMass Medical School, Worcester, Massachusetts, United States of America; 2 Department of Biology, McGill University, Montreal, Canada; 3 Department of Biological Sciences, University of Quebec at Montreal, Montreal, Canada; University of California San Diego, UNITED STATES

## Abstract

The regulation of cell migration is essential to animal development and physiology. Heparan sulfate proteoglycans shape the interactions of morphogens and guidance cues with their respective receptors to elicit appropriate cellular responses. Heparan sulfate proteoglycans consist of a protein core with attached heparan sulfate glycosaminoglycan chains, which are synthesized by glycosyltransferases of the exostosin (EXT) family. Abnormal HS chain synthesis results in pleiotropic consequences, including abnormal development and tumor formation. In humans, mutations in either of the exostosin genes *EXT1* and *EXT2* lead to osteosarcomas or multiple exostoses. Complete loss of any of the exostosin glycosyltransferases in mouse, fish, flies and worms leads to drastic morphogenetic defects and embryonic lethality. Here we identify and study previously unavailable viable hypomorphic mutations in the two *C*. *elegans* exostosin glycosyltransferases genes, *rib-1* and *rib-2*. These partial loss-of-function mutations lead to a severe reduction of HS levels and result in profound but specific developmental defects, including abnormal cell and axonal migrations. We find that the expression pattern of the HS copolymerase is dynamic during embryonic and larval morphogenesis, and is sustained throughout life in specific cell types, consistent with HSPGs playing both developmental and post-developmental roles. Cell-type specific expression of the HS copolymerase shows that HS elongation is required in both the migrating neuron and neighboring cells to coordinate migration guidance. Our findings provide insights into general principles underlying HSPG function in development.

## Author Summary

During animal development, cells and neurons navigate long distances to reach their final target destinations. Migrating cells are guided by extracellular molecular cues, and cellular responses to these cues are regulated by heparan sulfate proteoglycans. Heparan sulfate proteoglycans are proteins with long heparan sulfate polysaccharide chains attached. Here we identify and study previously unavailable viable mutants that disrupt the elongation of the heparan sulfate chains in the nematode *C*. *elegans*. Our analysis shows that these HS-chain-elongation mutations affect the development of the nervous system as they result in misguided migrations of neurons and axons. Furthermore, we find that heparan sulfate chain elongation occurs in numerous cell types during development and that the coordinated production of heparan sulfate proteoglycans, in both the migrating cell and neighboring tissues, ensures proper migration. Our findings highlight the critical roles of heparan sulfate proteoglycans in nervous system development and the evolutionary conservation of the molecular mechanisms driving guided migrations.

## Introduction

Cell migration is key to animal development and physiology. To reach their targets, migrating cells rely on guidance factors and morphogens, which can be regulated by heparan sulfate proteoglycans (HSPGs) [1]. HSPGs are cell-surface or extracellular proteins characterized by the attachment of heparan sulfate (HS) polysaccharide chains to the extracellular domain of their core protein [2]. HSPGs interact with molecules at the cell surface and in the extracellular matrix via both their HS chains and core proteins, and can function as co-factors that regulate the distribution of morphogens and that modulate the interactions between extracellular ligands and their receptors [1, 3]. HSPGs have been shown to be part of multiple signaling pathways across species and to be key to multiple developmental events, including those elicited by guidance cues such as Slit and Netrin, and morphogens such as Hhg, FGF, Sonic Hedgehog, Wnts, and BMPs [1, 2, 4, 5].

The importance of HSPGs during animal development has been extensively studied using mutations that disrupt individual HSPG core proteins. A number of HSPGs have been characterized in *C*. *elegans* using mutations that affect specific *core proteins*, such as mutations in *sdn-1*/syndecan, *lon-2*/glypican, *cle-1*/collagen type XVIII, *unc-52*/perlecan, *gpn-1/*glypican, and *agr-1/*agrin [6–26]. These studies have uncovered precise roles of individual HSPGs in morphogenesis and nervous system development. For instance, loss of *cle-1*/collagen type XVIII leads to synaptic defects at neuromuscular junctions, as well as specific neuronal cell and axon guidance defects [6, 7]; *unc-52/*perlecan promotes ectopic presynaptic bouton growth and affects the 4° dendritic branching of the neuron PVD [24, 25]; *sdn-1*/syndecan mutants exhibit a number of neuronal cell and axon guidance defects [12–14, 21]; and *lon-2/*glypican is important for cell and axon guidance [8, 12, 17, 21], particularly for netrin-mediated guidance events [8, 13]. Moreover, studies where two or three HSPG core proteins have been simultaneously mutated in double and triple mutants demonstrated that the combined actions of precise HSPGs ensure proper guidance of neurons and axons during development [8, 12, 13, 21]. All these analyses of mutations affecting specific HSPG core proteins have been instrumental to address the roles of HSPGs in development. However, mutations that remove particular HSPG core proteins inevitably also remove the HS chains that would have been attached to the missing core proteins. Therefore, the phenotypic consequences of such mutations in HSPG core proteins can be due to the absence of either the core protein or the HS chains, or both, making it difficult to extract the functional contribution of the HS chains *per se* with such analysis of HSPG core protein mutants.

To address the roles of the HS chains that are attached to HSPG core proteins, various mutations that affect HS chain biosynthesis have been analyzed [1]. HS chains are linear glycosaminoglycan (GAG) polysaccharides composed of alternating repeats of D-glucuronic acid (GlcA) and N-acetylglucosamine (GlcNAc) [27]. HS chain biosynthesis in the Golgi apparatus can be divided into three phases: (1) initiation, (2) elongation, and (3) chemical modifications.

First, a HS chain is *initiated* by the addition of a tetrasaccharide linker (synthesized by the step-wise addition of a xylose residue, a galactose residue, a galactose residue, and a GlcA residue) on a specific Ser residue of the HSPG core protein. This initiation step is catalyzed by a set of four initiation enzymes encoded by the glycosyltransferases genes *sqv-6*, *sqv-3*, *sqv-2*, and *sqv-8* in *C*. *elegans* [28–30]. Mutations in these four initiation genes have been characterized, revealing important morphogenetic roles in embryogenesis and vulva development [28, 29, 31]. However, these four initiation enzymes add the same tetrasaccharide linker also to the core proteins of chondroitin sulfate proteoglycans (CSPGs), as they also catalyze the initiation of chondroitin sulfate (CS) chains. Thus, specific roles for HS chains cannot be addressed in these mutants in which the initiation of both HS and CS chains is affected, with phenotypes resulting from the combined disruption of both HSPGs and CSPGs.

Once *initiated*, the second phase of HS biosynthesis is the *elongation* of HS chains. HS chain elongation is catalyzed by the HS copolymerase, a heterodimer composed of two glycosyltransferases of the EXT family. HS chain *elongation* has been shown to be crucial to animal development across metazoans, as its dysfunction results in pleiotropic consequences including abnormal morphogenesis and tumor growth. In *C*. *elegans*, null mutations in the exostosin glycosyltransferases *rib-1* and *rib-2* are embryonic lethal, indicating that HS elongation is essential for morphogenesis [32–34]. In *Drosophila*, null mutations in the exostosin genes *tout-velu*, *brother of tout-velu* and *sister of tout-velu* are lethal, and loss of their function leads to severe patterning defects with abnormal morphogen signaling in many developmental contexts [35–39]. For example, *tout-velu* mutants exhibit a lack of diffusion of Hh in the wing imaginal disc [35]. In zebrafish, mutations in EXT family members *ext2* (dackel) and *extl3* (boxer) are also lethal [40]. In mice, complete loss of function of EXT1 induces defective gastrulation and embryonic lethality [41]. Mutations in glycosyltransferases genes EXT1 and EXT2 in humans result in a dominant disorder called hereditary multiple exostoses, characterized by cartilage-capped skeletal tumors known as osteochondromas, which results in skeletal abnormalities and short stature [42–47]. Although the osteochondromas are most often benign tumors, malignant transformation into chondrosarcomas or osteosarcomas occurs in ~2% of HME patients [48]. However, their exact roles in bone development and homeostasis are not well understood.

Given the lethality associated with the complete loss of HS elongation across species, genetic analysis of such mutations has been possible for early developmental roles or for conditions where the gene function is only partially lost. For instance, conditional knockouts have been used to study later developmental roles of the HS copolymerase in mice [49, 50], and partially maternally rescued mutants (displaying partial phenotypes) have been studied in zebrafish [40] and in *C*. *elegans* [21, 32–34]. Indeed, *C*. *elegans* deletion mutations in the HS copolymerase genes *rib-1* and *rib-2*, namely *rib-1(tm516* or *ok556)* and *rib-2(tm710* or *qa4900)* die as embryos: homozygous mutant progeny from a homozygous mutant mother (animals genotypically m^-/-^ z^-/-^, where “m” and “z” indicate the **m**aternal and **z**ygotic genotypes, respectively), all die as embryos. In contrast, first generation homozygous mutant animals from a heterozygous mother (that is to say animals that are genotypically *rib-1*^m+/- z-/-^ or *rib-2*^m**+**/- z-/-^) are “maternally rescued”; they complete development and become adults, due to wild-type gene product inherited from their heterozygous mothers (**Table 1**) [32–34]. Such adult *rib-1(tm516)*^m+/- z-/-^ and *rib-2(qa4900)*^m+/- z-/-^ animals are largely but not completely maternally rescued, as they display locomotion defects and cannot lay eggs normally, becoming filled with their dead m^-/-^ z^-/-^ progeny [32–34]. It has been shown that one quarter of *rib-1(tm516)*^m+/- z-/-^ maternally rescued animals exhibit axon guidance defects for the neuron HSN, while no HSN defect was seen in *rib-2(tm710)*^m+/- z-/-^ maternally rescued animals [21, 32]. Thus, a thorough phenotypic analysis of *rib-1* and *rib-2* deletion mutants has been limited by both (a) the early embryonic lethality of *rib-1*^m-/- z-/-^ and *rib-2*^m-/- z-/-^ mutants, where later developmental stages cannot be examined, and (b) the presence of wild-type maternal product in *rib-1*^m+/- z-/-^ and *rib-2*^m+/- z-/-^, which profoundly rescues development, masking HS functional requirements [21, 32–34]. The function of HS elongation in *C*. *elegans* has also been examined by RNAi knockdown of *rib-1* and *rib-2*, where the HSN axon guidance defects were more penetrant than in maternally rescued animals [17]. Yet, developmental defects are likely partially penetrant as there is no phenocopy of embryonic lethality by RNAi knockdown of *rib-1* and *rib-2* [17]. Thus, the availability of partial loss-of-function mutations for the genes *rib-1* and *rib-2* would allow, if viable, a systematic study of developmental roles for HS *elongation* during the development of the nervous system in *C*. *elegans*.

**Table 1:** Phenotypic comparison of *rib-1* and *rib-2* mutant alleles.

Allele	Molecular Lesion	Overall phenotype of animals		Reference
		Homozygous M-/- Z-/-	Maternally rescued M+/- Z-/-	
*rib-1 (tm516)*	486 bp deletion and 32 bp insertion	99% of embryos die 1% die as first stage larvae	Complete development to adulthood 54% adults defective for egg-laying	[32]
*rib-1 (ok556)*	905 bp deletion	Embryos and early larvae die	Viable in this generation (no further information available)	[33]
*rib-1 (qm32)*	Stop to Lys	14% viable, become adults 100% of adults are defective for locomotion and egg-laying 32% of embryos die 80% of hatchlings die as larvae	Fully rescued larvae and adults, no lethality Wild-type development, locomotion and egg-laying	[54] This study
*rib-2 (qa4900)*	511 bp deletion	97% embryos die 3% remaining morphologically abnormal and die in first larval stage	Adults defective for locomotion and egg-laying	[34]
*rib-2 (tm710)*	1306 bp deletion	Embryos die	Viable in this generation (no further information available)	[32, 33]
*rib-2 (qm46)*	R434Q	63% viable, become adults 100% of adult are defective for locomotion and egg-laying 15% of embryos die 26% of hatchlings die as larvae	Fully rescued larvae and adults, no lethality Wild-type development, locomotion and egg-laying	[54] This study
*rib-1 (qm32); rib-2 (qm46)*	Stop to Lys R434Q	100% embryos die	Complete development to adulthood, but 100% adults are defective for locomotion and egg-laying	This study

After HS chain elongation, the third phase of HS biosynthesis is the chemical *modification* of HS chains by modifying enzymes such as epimerases and sulfotransferases [1]. Research in *C*. *elegans* has elegantly addressed the requirements for HS chain *modifications* in nervous system development. Specific roles in the guidance of neuronal cell and axon migrations, including the modulation of distinct guidance cues, have been uncovered using mutations in the HS modifying enzymes epimerase *hse-5* and in the sulfotransferases *hst-2*, *hst-3*.*1*, *hst-3*.*2*, and *hst-6* [9–12, 14, 15, 18–22, 26]. For instance, in the contexts of PVQ axon guidance and D-type motoraxon guidance, *slt-1/*Slit signaling acts in the same pathway as *hse-5* and *hst-6*, suggesting HSPGs modified by *hse-5* and *hst-6* may function with *slt-1/*Slit to guide the axons of both PVQ and motorneurons [10]. In addition, ectopic hypodermal expression of *hst-6* disrupts the guidance of the axon of the motorneuron DB7, and is dependent upon both *lon-2/*glypican and *slt-1/*Slit, suggesting that ectopic hypodermal 6O-sulfated LON-2*/*glypican may disrupt axon guidance through impacting *slt-1/*Slit signaling [11]. Also, interactions between HS modification enzymes and ephrin and integrin signaling have also been investigated in the contexts of PVQ and motoraxon guidance [10]. In contrast, whereas the core protein LON-2*/*glypican has been shown to function in netrin-mediated guidance [8], it remains to be determined which specific HS chain modifications may be important for the *unc-6/*Netrin signaling pathway.

Collectively, all of this remarkable prior work on the roles of HSPGs for nervous system development in *C*. *elegans*, targeting either *core* proteins or HS chain chemical *modifications*, has yielded a view in which sets of specific HSPGs, with distinct HS chemical modification patterns, interact with specific guidance pathways and contribute to context-dependent guidance decisions during the nervous system assembly. However, a general outlook on the functions of HSPGs in nervous system development by specifically disrupting the *presence* of HS chains across all HSPGs has been unavailable.

Here we report the identification of viable partial loss-of-function mutations in the two HS copolymerase glycosyltransferase genes *rib-1* and *rib-2* of *C*. *elegans*. We show that these mutations reduce HS levels and affect cell and axonal migration during nervous system development. We find that the HS copolymerase is expressed dynamically during morphogenesis, and that expression is sustained throughout life in specific cell types, consistent with HSPGs playing both developmental and post-developmental roles. Our findings indicate that proper axon guidance during the development of the nervous system requires coordinated HS chain elongation in both the migrating neuron itself and adjacent cells that secrete the extracellular matrix along which the growth cone extends. Our analysis highlights the functional importance of HSPGs during animal development.

## Results

### Identification of viable partial loss-of-function mutations of the genes *rib-1* and *rib-2* encoding the subunits of the HS copolymerase

The analysis of uncoordinated mutants of *C*. *elegans* has uncovered key genes underlying nervous system development and function over the past decades [51–53]. In order to identify new genes required for neuronal development, a genetic screen searching for maternally-rescued uncoordinated mutants was carried out by Hekimi *et al*. [54]. Briefly, an F2 clonal screen was performed, where P0 animals were mutagenized, from which individual F1 hermaphrodites were isolated and allowed to self-fertilize, followed by the selection of individual *wild-type F2* hermaphrodites that were allowed to self-fertilize, to finally screen for and select broods where *all the F3* animals were uncoordinated/abnormal [54]. Such a scheme allowed for the isolation of maternally *rescued* mutants, where F2 animals appear phenotypically normal even though they are genotypically homozygous mutant (m^+/-^ z^-/-^), due to wild-type gene product provided by the heterozygous F1 mother. Only in the next generation of homozygous mutants derived from a homozygous mutant mother, namely F3 m^-/-^ z^-/-^ animals, does the abnormal phenotype manifest. Several maternally rescued uncoordinated mutants were isolated in this screen [54–56], two of which, *qm32* and *qm46*, had similar phenotypes and were mutations in genes *mum-1* and *mum-3*, respectively *(mum* stands for maternal-effect uncoordinated and morphologically abnormal) [54].

*mum-1(qm32)* and *mum-3(qm46)* homozygous mutant animals from a homozygous mutant mother have severe defects: 32% of *mum-1(qm32)*^m-/- z-/-^ and 15% of *mum-3(qm46)*^m-/- z-/-^ die as embryos, and of the embryos that hatch, 80% of *mum-1(qm32)*^m-/- z-/-^ larvae and 26% of *mum-3(qm46)*^m-/- z-/-^ larvae die before reaching adulthood and display morphological abnormalities ([54]; summarized in **Table 1**). Fortunately, a proportion of *mum-1(qm32)*^m-/- z-/-^ and *mum-3(qm46)*^m-/- z-/-^ mutant animals are viable and complete development to become fertile adults [54], which allows the easy propagation of the homozygous mutant strains. In fact, 14% of *mum-1(qm32)*^m-/- z-/-^ animals and 63% of *mum-3(qm46)*^m-/- z-/-^ animals reach adulthood, all of which are uncoordinated and egg-laying defective [54]. Importantly, *mum-1(qm32)* and *mum-3(qm46)* mutations are recessive: animals genotypically heterozygous (z^+/-^) exhibit a wild-type phenotype, irrespective of the maternal genotype (m^-/-^ or m^+/-^) [54]. Moreover, *mum-1(qm32)*^m+/- z-/-^ and *mum-3(qm46)*^m+/- z-/-^ animals are *fully maternally rescued*: m^+/-^ z^-/-^ animals develop normally, display no lethality or morphological abnormalities, and become adults that are indistinguishable from the wild type, locomoting and laying eggs normally [54]. Worth noting, this maternal rescue effect is incomplete when both *mum-1(qm32)* and *mum-3(qm46)* mutations are combined. Indeed, first generation double homozygous mutant animals from doubly heterozygous mothers, i.e. *mum-1*^m+/- z-/-^; *mum-3*^m+/- z-/-^, develop into adults that are uncoordinated and egg-laying defective, becoming bloated with a brood of dead embryos that are *mum-1*^m-/- z-/-^; *mum-3*^m-/- z-/-^ double homozygous mutants. Thus, the double mutant *mum-1(qm32); mum-3(qm46)* could not be generated (we attempted to build it by several crossing schemes), as 100% of the animals die as embryos when there is no wild-type maternal contribution. Finally, the phenotype of both *mum-1(qm32)* and *mum-3(qm46)* over a deficiency is lethal, suggesting that the null phenotype of these two genes is lethal [54].

**Fig 1:**
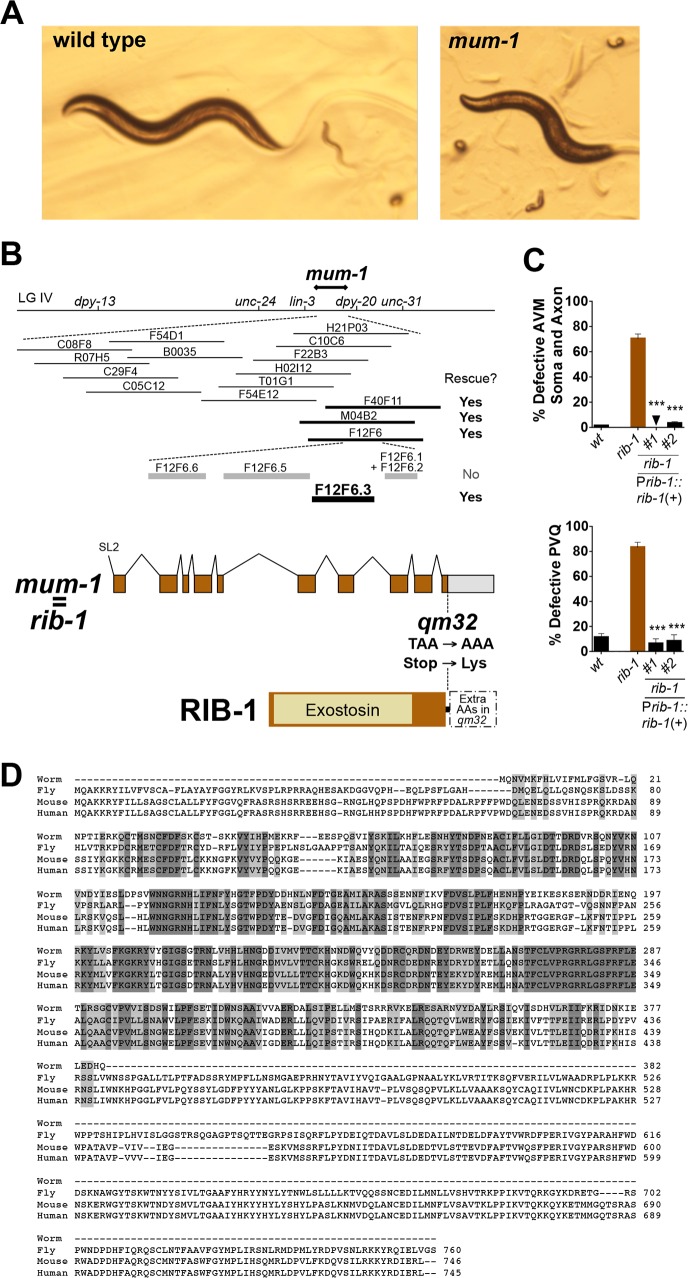
*mum-1* is a viable hypomorphic mutation of the gene *rib-1*, which encodes one of the two HS copolymerase subunits. **A.** Pictures of wild type and *mum-1/rib-1* mutant animals. Some of the *mum-1/rib-1* mutants die as embryos or misshapen larvae, and 14% of the animals reach adulthood and display defective locomotion and egg-laying, as described in [54]. **B.** Molecular identification of *mum-1(qm32)* as an allele of the gene *rib-1*. *mum-1* was previously mapped between *dpy-13* and *unc-31* on linkage group IV [54]. We narrowed down its genetic position by a combination of three-point and two-point mapping (results were *unc-24* 70/79 *mum-1* 9/79 *dpy-20*; 10 Dpy-20 non Mum-1/4320 F2s; and *lin-3* 6/7 *mum-1* 1/7 *dpy-*20; which placed *mum-1* between *lin-3* and *dpy-20*, between 4.98 and 5.07 cM, according to the 95% confidence intervals). Cosmids (thin lines) and PCR products (thick lines) encompassing the gene *rib-1(+)* rescued the morphological and uncoordination defects of the *mum-1* mutants. *mum-1(qm32)* is a missense mutation at base 39528 of cosmid F12F6 and changes the Stop into a Lys codon, which leads to a putative extension of 114 amino acids until the first in frame Stop codon. **C.** Rescue of the cell and axon guidance defects of the neuron AVM and axon guidance of the neurons PVQ of *mum-1(qm32)* with DNA corresponding to the genomic region of *rib-1(+)* (plasmid P*rib-1*::*rib-1*::*Venus*). See **S2 and S3 Tables** for sample sizes. Error bars are standard error of the proportion. Asterisks denote significant difference: *** *P* ≤ 0.001 (z-tests, P values were corrected by multiplying by the number of comparisons). **D.** An alignment of the predicted amino acid sequences of *C*. *elegans* RIB-1 (NP_502180.1) and its homologues from *D*. *melanogaster* (Ttv, NP_477231.1), *M*. *musculus* (EXT1, NP_034292.2), and *H*. *sapiens* (EXT1, NP_000118.2).

To uncover what genes were disrupted in the mutants *qm32* and *qm46* we determined the molecular identity of the lesions in these two mutants. We found that *qm32* and *qm46* are alleles of the genes *rib-1* and *rib-2*, respectively, as we demonstrate here. First, we narrowed down the genetic position of *mum-1(qm32)* by genetic mapping and then assayed cosmids corresponding to the genetic position of *mum-1(qm32)* for transformation rescue (**Fig 1B**). We found that cosmid F12F6 fully rescued the *mum-1(qm32)* mutants for uncoordination, egg laying defects, and abnormal larval morphology and lethality (**Fig 1A and 1B**). We tested PCR products corresponding to each of the genes located on this cosmid and found that a 9 kb PCR product containing the gene F12F6.3/*rib-1* fully rescued all of the above mutant phenotypes of *mum-1(qm32)* (**Fig 1A and 1B**). In addition, construct P*rib-1*::*rib-1(+)* completely rescued the cell and axon guidance defects of the neuron AVM, the axon guidance defects of the neurons PVQ in *mum-1(qm32)* mutants (**Fig 1C**), and their behavioral defects. We verified the predicted exon structure of the gene by sequencing cDNA clone yk187a9. We sequenced the genomic region of *rib-1* in *mum-1(qm32)* mutants and found that the *qm32* molecular lesion is a T to A base pair change at position 39528 of cosmid F12F6, which converts the Stop codon of *rib-1* into a Lys residue (**Fig 1B**). Thus, *mum-1(qm32)* corresponds to the gene previously known in the literature as *rib-1*, and we will refer to *mum-1(qm32)* as *rib-1(qm32)* from now on. The gene *rib-1* is homologous to exostosin family members mammalian EXT1 and *Drosophila tout-velu*, and thus encodes one of the two glycosyltransferases that compose the *C*. *elegans* HS copolymerase, responsible for HS chain elongation (see below, **Fig 1D**).

The *rib-1(qm32)* mutation does not affect the transcript levels of *rib-1* as assayed by RT-PCR (**S1B Fig**). Based on the sequence, it may result in the translation of an open reading frame present in the 3’UTR, which would possibly extend RIB-1 by 114 aa residues until the next in-frame Stop codon. The activity of the mutant RIB-1 protein in *rib-1(qm32)*, or of the complex in which it functions (see below), is affected by the mutation. The *rib-1(qm32)* mutation is fully recessive, fully maternally rescued, and is completely rescued by expression of wild-type transgenic copies of the gene [54] (this study), suggesting that the predicted protein extension diminishes RIB-1 activity in *rib-1(qm32)* mutants, rather than being neomorphic. Consistent with the notion that *qm32* is a partial loss-of-function mutation, the phenotype of *qm32* over a deficiency is more severe (i.e., lethal [54]), and the phenotype of null deletion alleles *rib-1(tm516)* and *rib-1(ok556)* is also more severe than that of *rib-1(qm32)*, as 100% of *rib-1(tm516)*^m-/- z-/-^ *and rib-1(ok566)*^m-/- z-/-^ animals die as embryos [32, 33], compared to 32% embryonic lethality in *rib-1(qm32)*^m-/- z-/-^ [54]. In sum, these data indicate that the mutation *qm32* is a hypomorphic mutation of the gene *rib-1*, where residual function allows 14% of the *rib-1(qm32)*^m-/- z-/-^ mutants to be viable and become uncoordinated and egg-laying defective adults.

**Fig 2:**
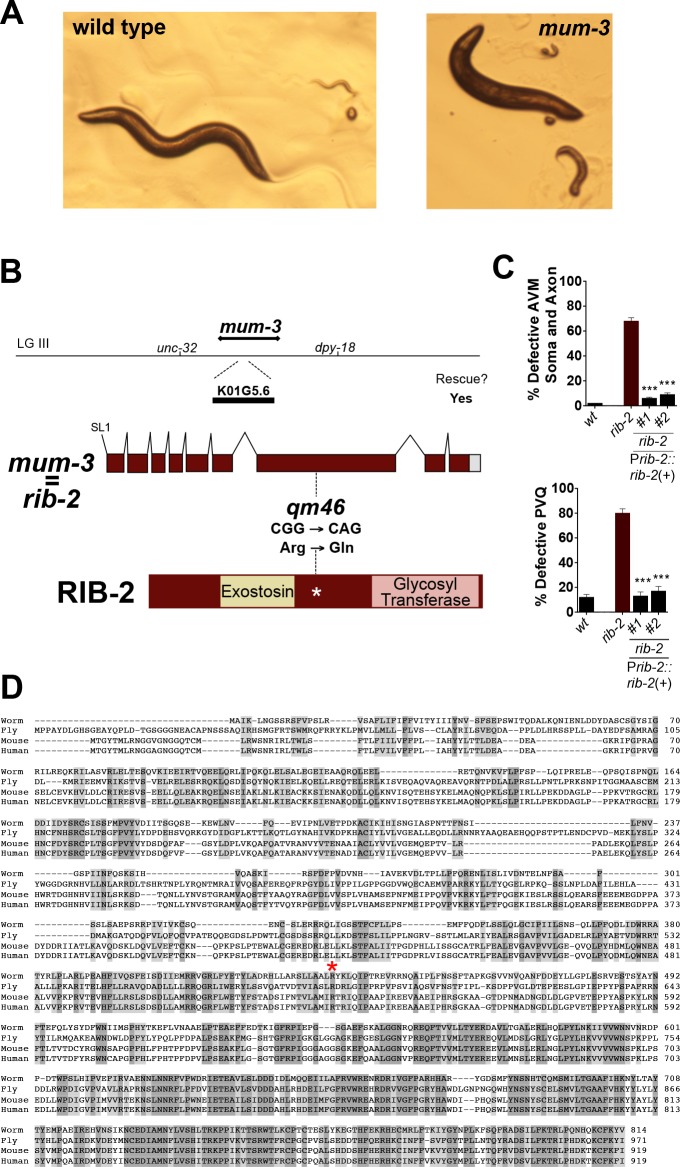
*mum-3(qm46)* is a viable hypomorphic mutation of the gene *rib-2*, which encodes the second HS copolymerase subunit. **A.** Pictures of wild type and *mum-3/rib-2* mutant worms. Some of the *mum-3/rib-2* mutants die as embryos or deformed larvae, and 63% animals reach adulthood and display defective locomotion and egg-laying, as described in [54]. **B.** Molecular identification of *mum-3(qm46)* as an allele of the gene *rib-2*. *mum-3* was previously mapped on linkage group III, between *unc-32* and *dpy-18* [54], which includes the second HSPG copolymerase gene *rib-2*. A PCR product containing the genomic locus of *rib-2(+)* rescued the morphological and uncoordination defects of the *mum-3(qm46)* mutants. *mum-3(qm46)* is a missense mutation at base 4366 on cosmid K01G5 that changes an Arg to a Gln at conserved residue 434, indicated by a red asterisk on the alignment (in **D**). **C.** Rescue of the cell and axon guidance defects of the neuron AVM and axon guidance of the neurons PVQ of *mum-3(qm46)* with DNA containing the genomic region of *rib-2(+)* (PCR product P*rib-2*::*rib-2*). See **S2 and S3 Tables** for sample sizes. Error bars are standard error of the proportion. Asterisks denote significant difference: *** *P* ≤ 0.001 (z-tests, P values were corrected by multiplying by the number of comparisons). **D.** An alignment of the predicted amino acid sequence of *C*. *elegans* RIB-2 (NP_499368.1) and its homologues from *D*. *melanogaster* (Botv, NP_523790.1), *M*. *musculus* (EXTL3, NP_061258.2), and *H*. *sapiens* (EXTL3, NP_001431.1).

**Fig 3:**
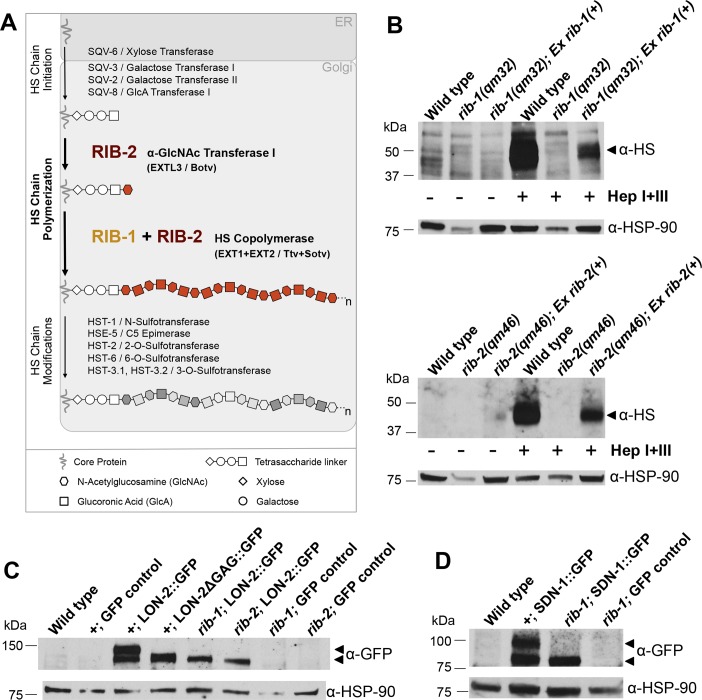
Loss of function of the genes *rib-1* and *rib-2* impairs HS synthesis. **A.** Schematic representation of the biochemical synthesis of HS chains onto HSPG core proteins in the Golgi. HS chain synthesis begins by the addition of a tetrasaccharide linker (xylose-galactose-galactose-glucuronic acid) onto specific Ser residues on the core proteins to serve as a primer for HS polysaccharide growth. HS chains are composed of repeating GlcA (square) and GlcNAc (hexagon) residues. RIB-2, like mammalian EXTL3 and *Drosophila brother of tout-velu (*Botv), catalyzes the first step of HS chain elongation by adding the first GlcNAc onto the tetrasaccharide linker. Then, a complex of RIB-1 and RIB-2 together, like mammalian EXT1 and EXT2, or *Drosophila tout-velu* (Ttv) and *sister of tout-velu* (Sotv), function as a heterodimer to extend the HS chains by adding subsequent GlcA and GlcNAc residues. Thus, RIB-2 serves two distinct roles of HS elongation: it catalyzes the start of the elongation and functions as a heterodimer with RIB-1 to elongate HS chains. HS chains are subsequently modified by a number of modifying enzymes. **B.** Western blot analysis of HS chains in *rib-1(qm32)*^m-/-z-/-^ mutants (top) and in *rib-2(qm46)* ^m-/-z-/-^ mutants (bottom). HS chains were detected with antibodies specific for HS epitope 3G10, which is detectable after cleavage by heparinase. For the three left lanes, protein extracts were not heparinase treated (control, indicated by “-“), and as expected no HS signal above background is detected. For the three right lanes, protein extracts were treated with heparinases I and III (Hep I+III, indicated by “+”). The HS signal is severely reduced in the *rib-1(qm32)*^m-/-z-/-^ and the *rib-2(qm46)*^m-/-z-/-^ mutants compared to wild type (N2), indicating that HS chain synthesis is strongly affected in these mutants. To rescue the HS level defect we used strains of *rib-1(qm32)*^m-/-z-/-^ and the *rib-2(qm46)*^m-/-z-/-^ mutants that carry the rescuing transgenes P*rib-1*::*rib-1(+)* or P*rib-2*::*rib-1(+)*, respectively, which restore, at least partly, HS levels. The rescue is partial likely because the rescuing transgene in each strain is harbored on an unstable non-integrated extrachromosomal array, which is lost during cell divisions and generations, and thus only ~10–20% of the animals of the strain actually carried the rescuing transgene when the worms were collected for analysis. Nonetheless, it is clear that HS biosynthesis is disrupted in the *rib-1(qm32)* and the *rib-2(qm46)* mutants, and their developmental and biochemical phenotypes can be rescued by reintroducing wild-type *rib-1(+)* and *rib-2(+)*, respectively. **C**. Western blot analysis of LON-2::GFP in the *rib-1(qm32)*^m-/-z-/-^ and *rib-2(qm46)*^m-/-z-/-^ mutants. The anti-GFP antibody detects two high molecular weight bands in extracts of transgenic animals expressing LON-2::GFP; as controls, these high molecular weight bands are absent in wild type and in three GFP controls that do not express LON-2::GFP (*lqIs4*, *rib-1(qm32);lqIs4*, and *rib-2(qm46);lqIs4*). In transgenic animals expressing a mutant version of LON-2 lacking the three HS attachment sites (LON-2ΔGAG::GFP; [67]), the detected band is smaller than LON-2::GFP. Similarly, the band detected in extracts of the *rib-1(qm32)* and *rib-2(qm46)* mutants expressing LON-2::GFP is smaller, indicating that the HS chains of glypican/LON-2 are affected in *rib-1* and *rib-2* mutants. **D.** Western blot analysis of SDN-1::GFP in the *rib-1(qm32)*^m-/-z-/-^ mutants. The anti-GFP antibody detects high molecular weight bands in extracts of transgenic animals expressing SDN-1::GFP; as controls, these high molecular weight bands are absent in the wild type N2 or the GFP control *rib-1(qm32); lqIs4*. In extracts of *rib-1(qm32)* mutants expressing SDN-1::GFP, the band detected is smaller, indicating that the HS chains of Syndecan/SDN-1 are affected in *rib-1* mutants. α-HSP-90 was used as a loading control.

The second *C*. *elegans* HS glycosyltransferase and subunit of the HS copolymerase that catalyzes HS chain elongation is encoded by the gene *rib-2*. Given the phenotypic similarities between the *mum-1/rib-1(qm32)* and *mum-3(qm46)* mutants and that the genetic mapping position of *mum-3(qm46)* corresponded to a chromosomal interval containing the gene *rib-2*, we determined whether *mum-3(qm46)* was an allele of *rib-2*. We tested a 5.6 kb PCR product containing *rib-2(+)* for rescue of *mum-3(qm46)* mutants and found that their defects in larval development, locomotion, and egg laying were fully rescued by this transgene (**Fig 2A and 2B**). In addition, construct P*rib-2*::*rib-2(+)* completely rescued the cell and axon guidance defects of the neuron AVM and axon guidance defects of the neuron PVQ in *mum-3(qm46)* mutants (**Fig 2C**), as well as their developmental and behavioral defects. We verified the predicted exon structure of the gene by sequencing cDNA clone yk3c1. We sequenced the genomic region of the gene *rib-2* in the *mum-3(qm46)* mutants and found that the *qm46* molecular lesion changes a G to A at position 4366 of cosmid K01G5. The *qm46* mutation results in an Arg to Gln amino acid substitution at conserved residue 434, which is near the exostosin domain in the 814 amino acid long RIB-2 protein (**Fig 2D)**. Thus, *mum-3(qm46)* corresponds to the gene previously known in the literature as *rib-2*, and we will refer to *mum-3(qm46)* as *rib-2(qm46)* from now on. The gene *rib-2* encodes the second glycosyltransferase subunit of the HS copolymerase and is most homologous to exostosin family members mammalian EXTL3 and *Drosophila brother of tout-velu*, and also fills the functional roles of EXT2 and *Drosophila sister of tout-velu* in *C*. *elegans* (**Figs 2D and 3A**).

Consistent with *qm46* being a missense mutation, the levels of *rib-2* transcript are comparable to wild type (**S1B Fig**). The *rib-2(qm46)* mutation is fully recessive, fully maternally rescued, and is completely rescued by expression of wild-type transgenic copies of the gene ([54]; this study), consistent with *rib-2(qm46)* being a partial loss-of-function mutation. Moreover, the phenotype of *qm46* over a deficiency is more severe (i.e., lethal [54]), and the phenotype of null deletion alleles *rib-2(tm710)*^m-/- z-/-^ and *rib-2(qa4900)*^m-/- z-/-^animals is also more severe as all embryos die [32–34]. In contrast, only 15% of *rib-2*(*qm46)*^m-/- z-/-^ animals die as embryos [54]. Taken together, *qm46* is a viable hypomorphic mutation in the gene *rib-2*, where residual function allows 63% of the *rib-2(qm46)*^m-/- z-/-^ mutants to be viable and become adults that are uncoordinated and egg-laying defective.

### HS levels are impaired in *rib-1(qm32)* and *rib-2(qm46)* mutants

The genes *rib-1* and *rib-2* each encode one of the two *C*. *elegans* HS glycosyltransferases that elongate HS chains (**Fig 3A)** [32, 57], which are composed of alternating GlcA and GlcNAc residues. After a tetrasaccharide linker has been synthesized on the HSPG core protein, the first step for HS chain *elongation* is the addition of a GlcNAc residue (**Fig 3A)** [57]. The addition of the first GlcNAc residue is catalyzed by RIB-2 in *C*. *elegans*, as demonstrated biochemically [57], similar to EXTL3 in mammals [58], and *Brother of tout-velu* in *Drosophila* [59]. HS chain elongation then proceeds by the repeated addition of disaccharide units of GlcA and GlcNAc (**Fig 3A)** [32]. This alternating addition of GlcA and GlcNAc is catalyzed by a heterodimer of RIB-1 and RIB-2, as demonstrated biochemically [32, 57], similar to EXT1 and EXT2 in mammals [60–63], and *Tout-velu* (Ttv) and the *Sister of tout-velu* in *Drosophila* [59].

Given the biochemically demonstrated roles of RIB-1 and RIB-2 [32, 57], the *rib-1(qm32)* and *rib-2(qm46)* mutations are expected to reduce HS chain elongation and result in decreased levels of HS in these mutants. To directly determine total HS levels in the *rib-1(qm32)*^m-/- z-/-^ and *rib-2(qm46)*^m-/- z-/-^ mutants, we performed Western blot analysis. For this, we extracted proteins from wild-type (N2) animals, *rib-1(qm32)*^m-/- z-/-^ and *rib-2(qm46)*^m-/- z-/-^ single mutants. We treated the protein extracts from these strains with a mix of heparinases I and III, and performed Western blot analysis using an antibody that specifically recognizes heparinase-digested HS chains (3G10, [64]). As expected, no signal above background was detected in untreated control samples (three left lanes, **Fig 3B**), compared to heparinase-digested samples (three right lanes, **Fig 3B**). Indeed, among the heparinase-digested samples, we found that compared to wild type, the HS content was severely reduced in *rib-1(qm32)* and *rib-2(qm46)* mutants (**Fig 3B**), confirming that the *rib-1(qm32)* and *rib-2(qm46)* mutations decrease HS biosynthesis, as predicted from their known biochemical functions [32, 57]. We examined whether the HS level reduction observed in these mutants could be rescued by transgenic expression of *rib-1(+)* and *rib-2(+)*, respectively. For this, we extracted proteins from a strain of *rib-1(qm32)*^m-/- z-/-^ mutants carrying a *rib-1(+)*-containing extrachromosomal array, and from a strain of *rib-2(qm46)*^m-/- z-/-^ mutants carrying a *rib-2(+)*-containing extrachromosomal array. We found that transgenic expression of *rib-1(+)* and *rib-2(+)* re-elevates HS levels in the *rib-1(qm32)*^m-/- z-/-^ and *rib-2(qm46)*^m-/- z-/-^ mutants, respectively (right most lanes in **Fig 3B**). HS levels rescue appears incomplete likely due to the fact that by the time that the worms populations from these strains were collected, only ~10–20% of the animals actually carried the rescuing transgene (extrachromosomal arrays are lost at some frequency during cell divisions and over the course of generations [65]). Nevertheless, our results clearly indicate that the alleles of *rib-1(qm32)*^m-/- z-/-^ and *rib-2(qm46)*^m-/- z-/-^ strongly reduce the levels of HS compared to the wild type and that copies of the wild-type transgene re-elevate the HS levels. These results support that the *rib-1(qm32)* and *rib-2(qm46)* mutations reduce the function of the genes which are important for HS elongation, consistent with prior biochemical demonstration of their function [32, 57].

Having examined how *rib-1(qm32)* and *rib-2(qm46)* mutations impact global HS levels, we further examined their consequences on two specific HSPGs, LON-2/Glypican and SDN-1/Syndecan. In these experiments, to detect LON-2/Glypican, we expressed green fluorescent protein (GFP)-tagged LON-2 (LON-2::GFP, [66]). We carried out Western blot analysis using anti-GFP antibodies as a probe. Whereas two high molecular weight bands corresponding to LON-2::GFP and HS-modified LON-2::GFP are detected in wild-type lysates, only one of the bands is detected in lysates of *rib-1*^m-/- z-/-^ and *rib-2*^m-/- z-/-^ mutants (**Fig 3C**), indicating that HS synthesis onto LON-2/Glypican is affected by loss of *rib-1* or *rib-2* function. Consistent with this interpretation, wild-type worms expressing a mutant version of LON-2 in which the HS attachment sites are mutated (LON-2ΔGAG::GFP, [67]) displayed a single high molecular weight band that migrated to the same molecular weight as LON-2::GFP when expressed in *rib-1(qm32)*^m-/- z-/-^ and *rib-2(qm46)* mutants^m-/- z-/-^ (**Fig 3C**). We next analyzed HSPG SDN-1/Syndecan using a similar strategy. We expressed GFP-tagged SDN-1/Syndecan (SDN-1::GFP, [14]) in wild-type and *rib-1(qm32)*^m-/- z-/-^ mutant worms, and probed for GFP in lysates of these worms. In wild-type lysates, we detected two high molecular weight bands corresponding to SDN-1::GFP and HS-modified SDN-1::GFP, but only detected a single band in lysates of *rib-1(qm32)*^m-/- z-/-^ mutants (**Fig 3D**), indicating that loss of *rib-1* impairs HS synthesis onto SDN-1/Syndecan. Thus, our results provide compelling evidence that *rib-1(qm32)*^m-/- z-/-^ and *rib-2(qm46)*^m-/- z-/-^ mutations drastically reduce HS content, consistent with these mutations impairing HS chain elongation. As a note, using fluorescence microscopy, we observed that the expression of LON-2::GFP in *rib-1(qm32)*^m-/- z-/-^ and *rib-2(qm46)*^m-/- z-/-^ mutants was similar to wild type, and that SDN-1::GFP was similar to wild type in *rib-1(qm32)*^m-/- z-/-^ mutants (**S2 Fig**).

### Cell and axonal migration is impaired in *rib-1(qm32)* and *rib-2(qm46)*

**Fig 4:**
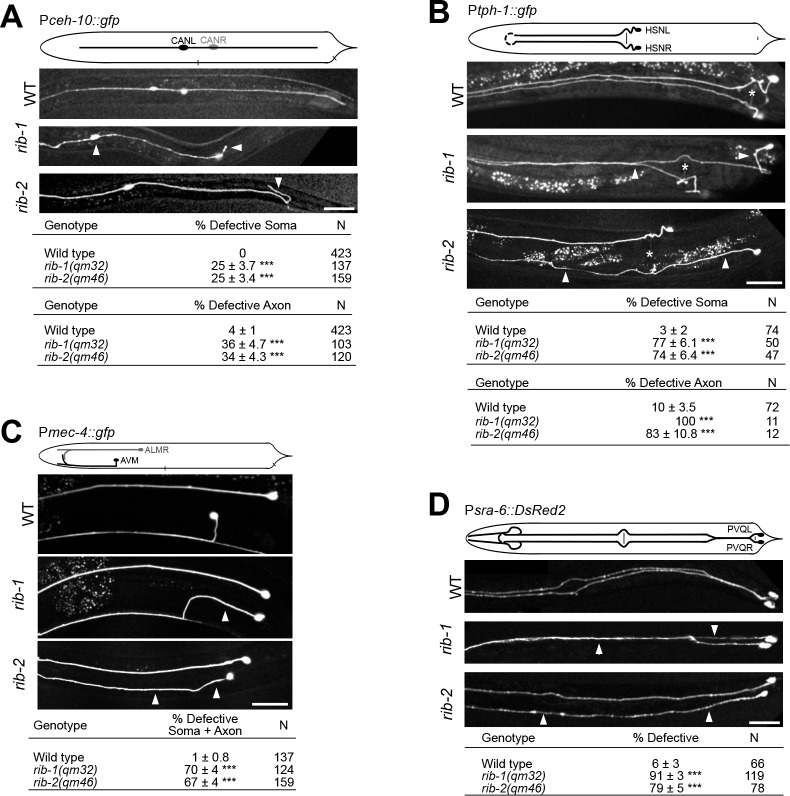
Neuronal migration defects in *rib-1(qm32)*^m-/-z-/-^ and *rib-2(qm46)*^m-/-z-/-^ mutants. **A.** Lateral views of animals expressing the transgene P*ceh-10*::*gfp* to visualize the CAN neurons (CANL and CANR). In the wild type, the soma of the CAN neuron is located laterally in the midbody region and its axon extends laterally along the antero-posterior axis. *rib-1(qm32)* and *rib-2(qm46)* mutants display defective CAN soma migration and are frequently observed in very anterior positions. In addition, the CAN axon in *rib-1* and *rib-2* mutants project in abnormal directions (arrowhead), or fail to extend fully in the posterior direction. The top table indicates CAN soma migration defects and the bottom table indicates axon guidance defects of the CAN neurons (axon guidance was only scored in animals that had normal soma migration). **B**. Ventral views of animals expressing the transgene P*tph-1*::*gfp* to visualize the HSN neurons (HSNL and HSNR). In wild type, the soma of HSN is located laterally just posterior to the vulva and its axon extends ventrally and projects along the ipsilateral side of the ventral nerve cord. *rib-1(qm32)* and *rib-2(qm46)* mutants display defective HSN soma positions and defective axons that extend laterally or project into the opposite side of the ventral nerve cord. The top table indicates HSN soma migration defects and the bottom table indicates axon defects of the HSN neurons (axon guidance was only scored in animals that had normal soma position). White asterisk indicates the position of the vulva. **C**. Lateral views of animals expressing the transgene P*mec-4*::*gfp* to visualize the AVM neuron. In the wild type, the AVM soma is located in the anterior midbody region and its axon projects ventrally to reach the ventral nerve cord and then extends anteriorly. *rib-1(qm32)* and *rib-2(qm46)* mutants display defective AVM soma (posterior to the vulva), as well as defective axons that project laterally instead of ventrally. The table indicates the sum of soma and axon defects of the AVM neuron (axon guidance was only scored in animals that had normal soma position). **D**. Ventral views of animals expressing the transgene P*sra-6*::*DsRed2* to visualize the PVQ neurons (PVQL and PVQR). In the wild type, the axon of PVQ extends along the ipsilateral side of the ventral nerve cord. *rib-1(qm32)* and *rib-2(qm46)* mutants display defective PVQ axons, including axons that extend laterally or project into the opposite side of the ventral nerve cord.

*rib-1(qm32)* ^m-/- z-/-^ and *rib-2(qm46)* ^m-/- z-/-^ mutants are uncoordinated and egg-laying defective. To gain insight into the impact of HS chain elongation on neuronal development, we set out to characterize the neuroanatomy of *rib-1(qm32)*^m-/- z-/-^ and *rib-2(qm46)*^m-/- z-/-^mutants. For this, we built strains of the *rib-1(qm32)*^m-/- z-/-^ and *rib-2(qm46* ^m-/- z-/-^ mutants carrying integrated transgenes to drive the expression of fluorescent proteins and allow the visualization of specific neurons (see **S9 Table**). We examined the nervous system of *rib-1(qm32)*^m-/- z-/-^ and *rib-2(qm46)*^m-/- z-/-^ adult viable mutants and found that the overall organization of the nervous system is grossly normal, as neuronal ganglia, axon fascicles and isolated neurons were generally well laid out. Examination with single-cell resolution revealed that numerous neuronal migrations are affected in both mutants. For instance, the CAN neuron cell body, which migrates from the head region towards the midbody region in wild-type animals, is frequently positioned too anteriorly or too posteriorly in both mutants (**Fig 4A**). Also, the HSN neuron cell body, which migrates from the tail region to the midbody region in wild type, is often located too posteriorly in both *rib-1(qm32)* and *rib-2(qm46)* mutants (**Fig 4B**). Moreover, the AVM neuron cell body is frequently located in the posterior of the body instead of being anterior to the vulva (**Fig 4C**). The penetrance and expressivity of these defects is similar in both mutants. Thus, loss of function of the genes *rib-1* or *rib-2* impairs the guidance of diverse neurons that undergo long-range migrations during development.

**Fig 5:**
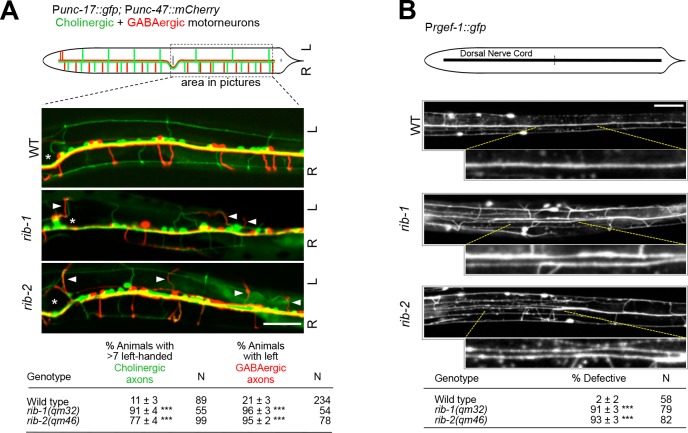
Defective motoraxon guidance in *rib-1(qm32)* and *rib-2(qm46)* mutants. **A.** Diagram depicts motoraxon commissures of cholinergic (green) and GABAergic (red) neurons that are visible in animals expressing the transgenes P*unc-17*::*gfp* and P*unc-47*::*mCherry*, respectively. Most cholinergic and GABAergic motoraxons extend along the right side (R) of the animal in the wild type. In fact, only seven cholinergic axons (green) and only two GABAergic axons (red) are present on the left side (L) of the animal. The three panels of fluorescent images below show the ventral views of only the posterior region (boxed on the diagram above) of animals expressing the two transgenes P*unc-17*::*gfp* and P*unc-47*::*mCherry*. The position of the vulva is indicated by an asterisk. For measuring the defects in animals, motoraxons were examined and recorded along the entire length of the animal, from the terminal bulb of the pharynx to the tail. In wild-type animals, the majority of motoraxons extend along the right side (R) of the animal: all but seven cholinergic axons (green) extend on the right side of the animal, and all but two GABAergic axons (red) extend on the right side. When more than seven cholinergic axons (green) were seen on the left side of a given animal, such animal was scored as defective. Similarly, when more two GABAergic axons (red) were seen on the left side, such an animal was scored as defective. *rib-1(qm32)* and *rib-2(qm46)* mutants display defective guidance of motorneuron axons of both cholinergic and GABAergic motorneuron axons, as more axons project along the left side (L) of the animal (arrowheads indicate misguided axons) compared to wild type. Rather than scoring each motoraxon individually, the data reported in the table is the percentage of animals with misguided axons. **B**. Dorsal views of animals expressing the transgene P*rgef-1*::*gfp* to visualize the dorsal nerve cord. The dorsal nerve cord runs as one tight fascicle in the wild type. In *rib-1(qm32)* and *rib-2(qm46)* mutants, the dorsal nerve cord is frequently split into two or more fascicles. Indicated area is enlarged in insets. Scale bars 20 μm. *** *P* ≤ 0.001 (z-tests, P values were corrected by multiplying by the number of comparisons).

We also found that axonal projections are defective in *rib-1(qm32)*^m-/- z-/-^ and *rib-2(qm46)*^m-/- z-/-^ single mutants. For example, the axon of the interneuron PVQ, which projects into the ipsilateral fascicle of the ventral nerve cord in the wild type, frequently projects in the contralateral fascicle or even laterally in *rib-1(qm32)* and *rib-2(qm46)* mutants (**Fig 4D**). Similarly, the axon of the motorneuron HSN, which projects ventrally and into the ipsilateral fascicle of the ventral nerve cord in the wild type, is misguided in the *rib-1(qm32)* and *rib-2(qm46)* mutants as it projects into the contralateral fascicle or laterally in these mutants (**Fig 4B**). These defects in HSN axon guidance are consistent with those reported for RNAi knockdown of *rib-1* and *rib-2*, as well as in maternally rescued *rib-1* animals [17, 32]. Another example is the axon of the mechanosensory neuron AVM, which extends ventrally towards the ventral nerve cord in the wild type, projects laterally in the mutants (**Fig 4C**). The axons of cholinergic and GABAergic motorneurons are also misguided in *rib-1(qm32)* and *rib-2(qm46)* mutants: contrary to the wild type, where most motorneuron axons exit the ventral midline on the right side to migrate along on the right side of the worm’s body wall, many motorneuron axons abnormally project to the left side in *rib-1(qm32)* and *rib-2(qm46)* mutants (**Fig 5A**). Finally, the dorsal nerve cord, which is composed of several motoraxons that run as a single fascicle in the wild type, is frequently defasciculated into several bundles in *rib-1(qm32)* and *rib-2(qm46)* mutants (**Fig 5B**). Thus, the guidance of numerous axons is disrupted upon loss of function of the genes *rib-1* and *rib-2*.

**Fig 6:**
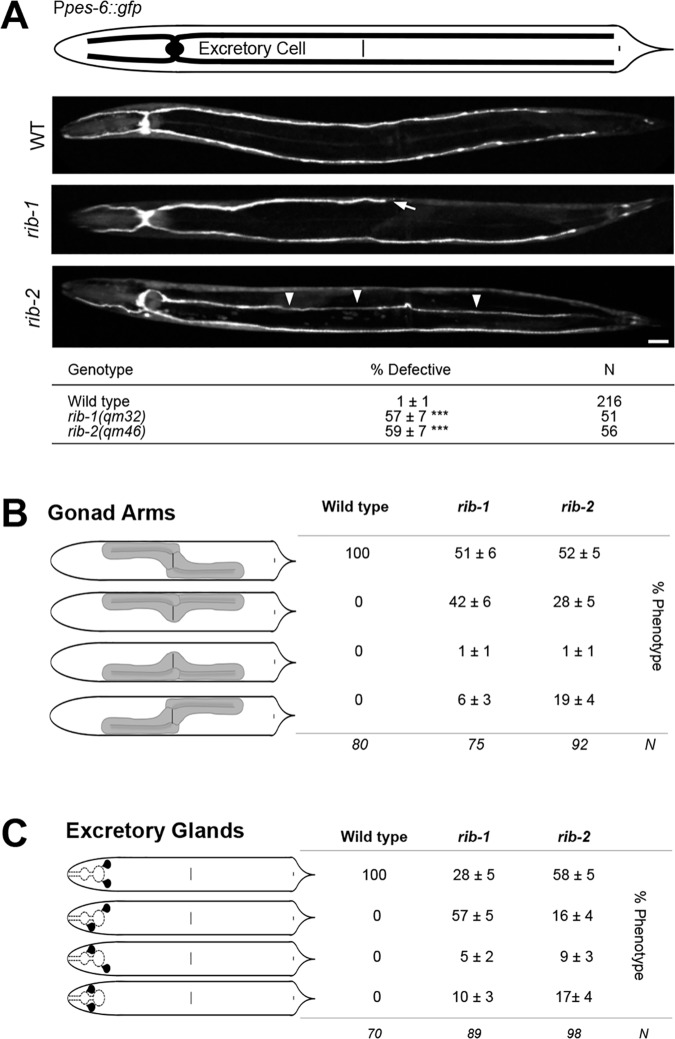
Other migration defects in *rib-1(qm32)*^m-/-z-/-^ and *rib-2(qm46)*^m-/-z-/-^ mutants. **A**. Ventral views of animals expressing the transgene P*pes-6*::*gfp* to visualize the canals of the excretory cell. In the wild type, the excretory cell extends four lateral canals, two that are anteriorly-directed along the sides of the head and two that are posteriorly-directed along the sides of the body. *rib-1(qm32)* and *rib-2(qm46)* mutants display defective excretory canals that can be too short (arrows), or extend ventrally or dorsally instead of laterally (arrowheads). Scale bar, 20 μm. Asterisk denotes significant difference: *** *P* ≤ 0.001 (z-tests, P values were corrected by multiplying by the number of comparisons). **B**. Diagrams of the gonad arms (dorsal view). In the wild type, the anterior arm of the gonad is located on the right side of the animal, and the posterior arm is located on the left side. Gonad arms are abnormally positioned in *rib-1(qm32)* and *rib-2(qm46)* mutants, where one or both gonad arms can lie on the opposite side of the animal. **C**. Diagrams of the excretory glands (dorsal view). In the wild type, the excretory glands are located just posterior of the terminal bulb of the pharynx. *rib-1(qm32)* and *rib-2(qm46)* mutants display abnormally located excretory glands, where one or both lie anterior of the terminal bulb.

In a similar way, the migration of mesodermal cells, which share guidance mechanisms with neurons [68], is defective in *rib-1(qm32)*^m-/- z-/-^ and *rib-2(qm46)*^m-/- z-/-^ single mutants. For instance, the canals of the excretory cell (two anterior and two posterior canals) run laterally in the wild type but are frequently too short or extend along the ventral or dorsal aspect of the body in *rib-1(qm32)* and *rib-2(qm46)* mutants (**Fig 6A**). Another example of misguided mesodermal cells in the *rib-1(qm32)* and *rib-2(qm46)* mutants is that of the distal tip cell (DTC), whose path determines the shape of the gonad. In wild-type animals, the anterior gonad arm is located on the right side of the animal, as the anterior DTC migrates along the right side, first anteriorly, then turning dorsally, and migrating back posteriorly towards the midbody region. Similarly, the posterior gonad arm is located on the left side, as the posterior DTC migrates posteriorly, turns dorsally and migrates back towards the midbody region. In *rib-1(qm32)* and *rib-2(qm46)* mutants, the sidedness of the gonad arms is often abnormal, with the anterior arm of the gonad on the left side of the animal and the posterior arm on the right side, or even having both gonad arms on the opposite side of the animal (**Fig 6B**). Lastly, *rib-1(qm32)* and *rib-2(qm46)* mutants display abnormal positioning of the excretory glands (**Fig 6C**). Thus, loss of function of the genes *rib-1* or *rib-2* disrupts the guidance of migrations of neuronal and mesodermal cells during development.

### The HS copolymerase is dynamically expressed during development

**Fig 7:**
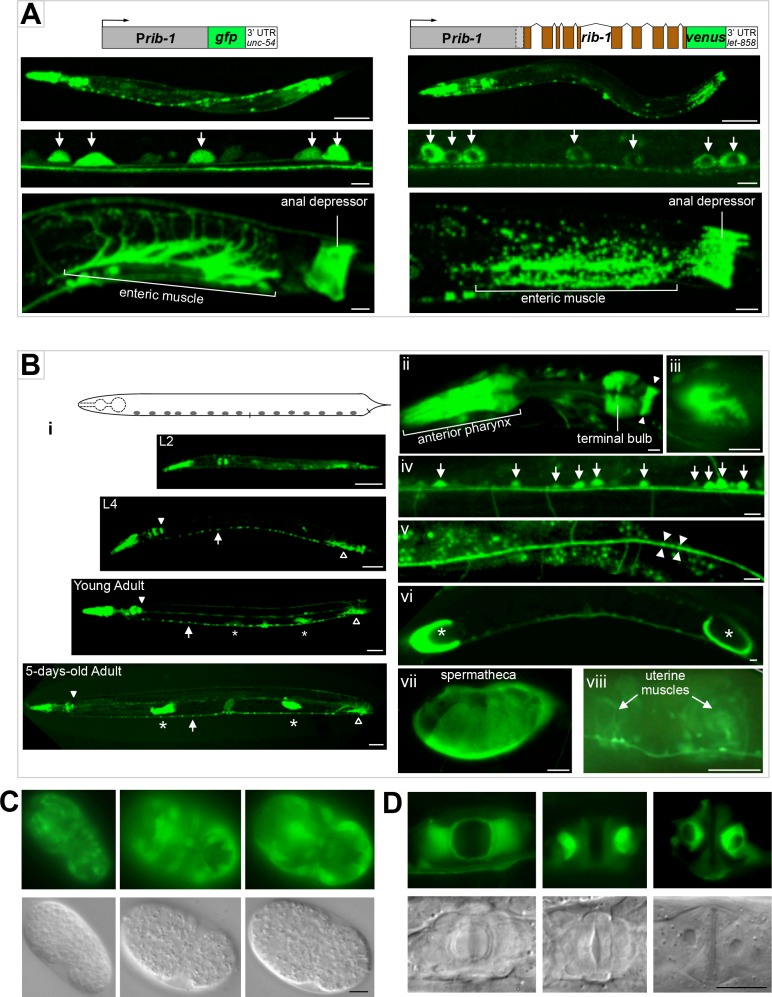
Dynamic expression of the HS copolymerase during development. **A.** *rib-1* transcriptional (P*rib-1*::*gfp*) and functional translational (P*rib-*1::*rib-1*::*venus)* reporters display similar expression patterns. Expression of *rib-1* is observed in numerous cell types, including in the pharynx, hypodermal cells, neurons (arrows), the enteric muscle and the anal depressor. The RIB-1::Venus signal is punctate, consistent with a Golgi localization. Scale bars: 50 μm in top panels of entire worms, and 5 μm in lower panels. **B.** Larval and adult expression of *rib-1*. (**i**) Throughout larval and adult stages, the expression of P*rib-1*::*gfp* is observed in the pharynx, including in the pharyngeal-intestinal valve (**ii**, closed arrowhead) and muscles of the anterior and terminal pharyngeal bulbs (**iii**, pharyngeal muscle pm6); in the enteric muscle (open arrowhead, see **A**); in the ventral nerve cord (arrows, **iv**, see **A**); in the dorsal nerve cord (**v**); in spermathecae (asterisks, **vi**, seen enlarged by an oocyte in **vii**); and in uterine muscles (**viii**). Scale bars, 50 μm in panels depicting entire worms, and 5 μm in the rest of the panels. **C**. Embryonic expression of *rib-1*. Views of comma stage embryos in GFP epifluorescence and DIC. The transcriptional reporter P*rib-1*::*gfp* is predominantly expressed in lateral and ventral epidermal cells that undergo major morphogenetic movements during dorsal intercalation and ventral enclosure of the embryo. Scale bar, 5 μm. **D.** *rib-1* is expressed during vulva development. Views of the developing vulva in fourth larval stage animals by GFP and DIC epifluorescence. P*rib-1*::*gfp* is expressed in epidermal cells of the developing vulva at a time when these cells undergo major rearrangements. Scale bar, 10 μm.

To gain insight into the roles of HSPGs during development, we determined the expression pattern of the HS copolymerase. For this, we designed a transcriptional fusion, P*rib-1*::*gfp*, between the upstream regulatory region of *rib-1* and *gfp*. Since *rib-1* is the second gene in a two-gene operon [69], we included the region upstream of the first gene in the operon, as well as the intergenic region of the operon that lies immediately upstream of *rib-1* (see Materials and Methods). Second, we constructed a translational fusion for *rib-1* using the same upstream regulatory region as for P*rib-1*::*gfp*, and fusing the coding region of *rib-1* with *venus*, a *gfp* variant that fluoresces in acidic cellular environments [70], to make the translational fusion P*rib-1*::*rib-1*::*venus*. We generated at least five transgenic lines for each of these two *rib-1* reporters and examined transgenic animals by fluorescence microscopy. We observed that the GFP signal from the transcriptional fusion P*rib-1*::*gfp* fills the cytoplasm of expressing cells, whereas the VENUS signal displays a punctate cytoplasmic pattern in cells expressing the translational fusion P*rib-1*::*rib-1*::*venus*, consistent with RIB-1 being localized to the Golgi apparatus (**Fig 7A**). Moreover, we found that both the transcriptional and translational fusions have a very similar spatial and temporal expression pattern during development: expression was visible in neurons, hypodermal cells, muscles of the digestive system, and reproductive tissues (**Fig 7A**). Importantly, we found that the translational fusion P*rib-1*::*rib-1*::*venus* is functional, as it fully rescues the defective locomotion, egg-laying, morphology, and axon guidance of *rib-1(qm32)*^m-/- z-/-^ mutants. Our observations indicate that the observed expression pattern of the translational reporter and of the very similarly expressed transcriptional reporter are functionally relevant and largely reflect sites of endogenous *rib-1* expression.

Since the transcriptional and translational *rib-1* fusions have similar expression patterns, we used P*rib-1*::*gfp*, which has a stronger expression level, to characterize the expression pattern of *rib-1* in more detail. We found that P*rib-1*::*gfp* is broadly expressed in ectodermal and mesodermal cells during embryogenesis. A salient feature of the *rib-1* expression pattern is that it is very dynamic in hypodermal cells during development. In embryogenesis, P*rib-1*::*gfp* is detected along the entire layer of hypodermoblasts that surrounds the gastrulating embryo at about 200 minutes after fertilization. By the early comma stage of embryogenesis, P*rib-1*::*gfp* is expressed at high levels in hypodermal cells of the elongating embryo (**Fig 7C**), including hypodermal cells extending ventrally during ventral closure and in the two rows of dorsal hypodermal cells undergoing dorsal intercalation. Following these embryonic morphogenetic events, expression of P*rib-1*::*gfp* in the hypodermal cells of the body wall is no longer visible during larval and adult stages, except for seam cells undergoing fusion during larval development. Also, hypodermal cells of the developing vulva express P*rib-1*::*gfp* (**Fig 7D**), at a low expression level in L3 larvae and at a stronger level in L4 larvae and just molted young adults, and vanishing in vulval cells in the adult. These dynamic expression patterns in cells undergoing dramatic changes during morphogenesis suggest a potential for rapidly changing needs for particular HSPGs in specific tissues at different time points during development.

The nervous and digestive systems express P*rib-1*::*gfp* stably and continuously from embryogenesis throughout adulthood. Strong and sustained expression is seen in motorneurons, interneurons, sensory neurons (including AVM), neurons in the head and tail ganglia, with the GFP signal filling axons running along the ventral and dorsal nerve cords, commissures, and sublaterals. Expression in neurons of the ventral nerve cord and of the head ganglia is visible in 1.5-, 2-, and 3-fold embryos, and persists into adulthood (**Fig 7A and 7B**). Strong expression of P*rib-1*::*gfp* is also observed in the pharynx from the 2-fold stage of embryogenesis onwards and remained strong in adults (procorpus, metacorpus, terminal bulb, grinder, and pharyngeal-intestinal valve). The anal depressor, the anal sphincter, the two enteric muscles, the spermathecae and the uterine muscles maintain expression in adults (**Fig 7B**). The continued expression of *rib-1* in the nervous, digestive and reproductive systems suggests that HSPGs play not only developmental, but also post-developmental roles in these cell types.

### HSPG synthesis in multiple cell types contributes to axon guidance

**Fig 8:**
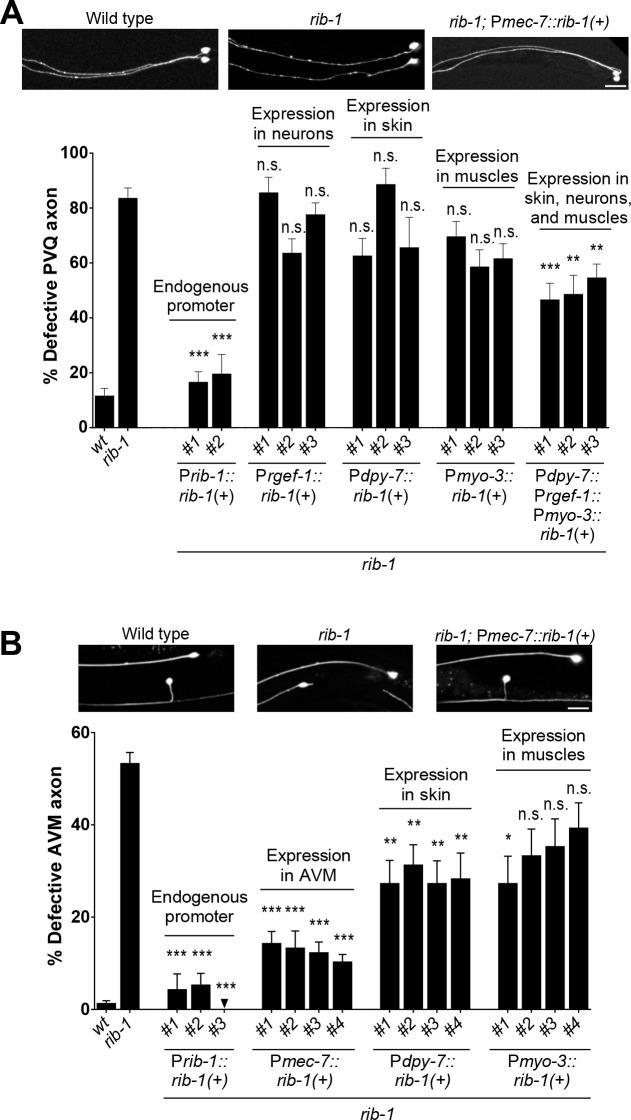
HS chain elongation in multiple cell types contributes to axon guidance. **A.** The defects in the guidance of the PVQ axons in *rib-1(qm32)*^m-/-z-/-^ mutants are rescued by the combined expression of *rib-1*(+) in the hypodermis (under the heterologous promoter P*dpy-7)*, neurons (under the heterologous promoter P*rgef-1)*, and muscles (under the heterologous promoter P*myo-3)*. **B**. The defects in the guidance of the AVM axon in *rib-1(qm32)*^m-/-z-/-^ mutants are rescued by expression of *rib-1(+)* in the migrating neuron itself (under the heterologous promoter P*mec-7*) or by expression in the hypodermis (under the control of the heterologous promoter P*dpy-7*). Scale bar, 20 μm. Error bars are standard error of the proportion. Asterisks denote significant difference: *** *P* ≤ 0.001, ** *P* ≤ 0.01, * *P* ≤ 0.05 (z-tests, P values were corrected by multiplying by the number of comparisons). ns, not significant. See **S4** and **S5 Tables**.

A prominent site of expression of the HS copolymerase is the nervous system, including during axon migration in embryonic and larval development (**Fig 7**), and disruption of the HS copolymerase in *rib-1(qm32)*^m-/- z-/-^ or *rib-2(qm46)*^m-/- z-/-^ mutants leads to numerous axon guidance defects (**Fig 4 and Fig 5**). To determine in which cells HS production is required for axon guidance, we provided *rib-1(qm32)*^m-/- z-/-^ mutants with wild-type copies of *rib-1(+)* in subsets of cells and assessed rescue of the PVQ axon guidance defects. The axon of PVQ extends along the ventral nerve cord during embryogenesis, following the path of other axons, and is in proximity with the hypodermis and body wall muscles. We built constructs to express *rib-1(+)* in neurons including PVQ (using the heterologous promoter P*rgef-1*), in the hypodermis (using the heterologous promoter P*dpy-7*), or in body wall muscles (using the heterologous promoter P*myo-3*). We then generated transgenic *rib-1(qm32)* worms expressing *rib-1(+)* in these tissues and analyzed PVQ axon guidance. As a control, we determined that expression of *rib-1(+)* under its own promoter completely rescued the guidance defects of the PVQ axon (**Fig 8A**). Targeted expression of *rib-1(+)* only in neurons, only in the hypodermis, or only in body wall muscles did not rescue the *rib-1* mutant PVQ axon guidance defects. However, co-expression of *rib-1(+)* simultaneously in the hypodermis, neurons, and body wall muscles led to a significant rescue of these defects (**Fig 8A**), suggesting that HSPGs derived from specific cell types together contribute to proper PVQ axon guidance. The rescue of the PVQ axon was strong but incomplete, possibly due to the inappropriate *rib-1* expression level or timing under these heterologous promoters. Nonetheless, expressing *rib-1* simultaneously in these three tissues yielded a significant rescue of the PVQ defects, indicating a simultaneous functional requirement for HSPGs in distinct cell types to regulate the guidance of the PVQ axon.

We next turned to elucidating the spatial requirements of HS biosynthesis for guidance of the mechanosensory neuron AVM. During the first larval stage, the AVM axon pioneers its own ventral migration through a basement membrane along the body wall, sandwiched between the hypodermis and body wall muscles. We expressed *rib-1(+)* in the hypodermis (using the heterologous promoter P*dpy-7*), in body wall muscles (using the heterologous promoter P*myo-3*), or in AVM itself (using the heterologous promoter P*mec-7)* in *rib-1(qm32)*^m-/- z-/-^ mutants, and analyzed AVM axon guidance. As a control, we determined that expression of *rib-1(+)* under its own promoter completely rescued the guidance defects of the AVM axon (**Fig 8B**). We found that the AVM axon guidance defects of *rib-1* mutants were rescued by expression of *rib-1(+)* in AVM itself, or by expressing *rib-1(+)* in the hypodermis (**Fig 8B**). These results suggest that HSPGs derived from both AVM and the hypodermis crucially impact AVM axon guidance. Taken together, our results support the notion that HSPGs synthesized in distinct cell types coordinate guided axonal migration during development.

### HS chain elongation is required for *unc-6*/Netrin-mediated axon guidance

**Fig 9:**
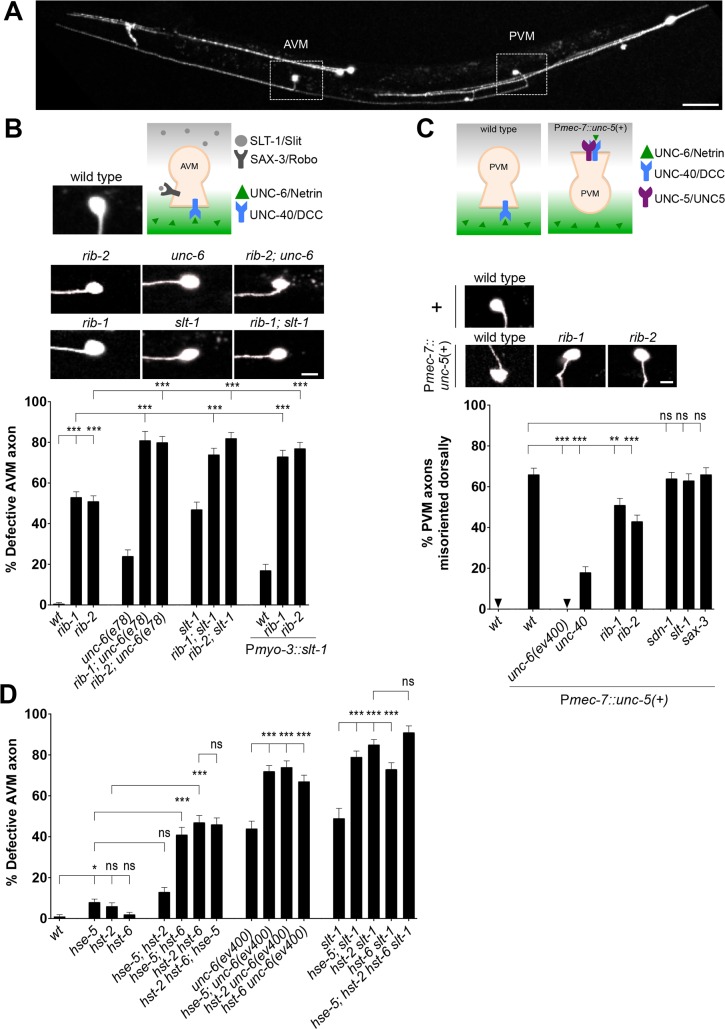
HS chain elongation is required for *unc-6*/Netrin-signaling in axon guidance. **A.** We visualized the morphology of the AVM and PVM axons using the transgene P*mec-4*::*gfp*. Scale bar, 50 μm. **B**. During the first larval stage of wild-type *C*. *elegans*, the pioneer neuron AVM extends ventrally along the body wall until it reaches the ventral nerve cord. Its migration results from the combined attractive response to UNC-6/Netrin (secreted at the ventral midline) via the UNC-40/DCC receptor, and the repulsive response to SLT-1/Slit (secreted by the dorsal muscles) via its SAX-3/Robo receptor. Loss of *rib-1* or *rib-2* combined with defective *unc-6*/Netrin function using the hypomorphic allele of *unc-6(e78)*, results in more severely affected AVM guidance. Hypomorphic allele *unc-6(e78)* was used here because the double mutants of *rib-1(qm32)* or *rib-2(qm46)* with the null allele *unc-6(ev400)* died as embryos. Combining mutations *rib-1(qm32)*^m-/-z-/-^ or *rib-2(qm46)*^m-/-z-/-^ with conditions where *slt-1*/Slit signaling is disrupted, either with the presumptive null allele *slt-1(eh15)* or in animals misexpressing *slt-1* in all body wall muscles (using a P*myo-3*::*slt-1* transgene), leads to more severe AVM defects that in either single condition alone. That the AVM axon guidance defects of mutants in both key guidance pathways, *slt-1* and *unc-6*, are worsened by loss of *rib-1* or *rib-2* is consistent with the notion that HS chains function in both of these pathways, and/or function in yet another unidentified pathway. Scale bar, 5 μm. **C.** *unc-6*/Netrin signaling via the *unc-5*/UNC5 receptor requires functional HS chains. The pioneer axon of PVM normally migrates ventrally; however, upon misexpression of *unc-5*/UNC5 in PVM using the transgene P*mec-7*::*unc-5*, the axon of PVM projects dorsally in an *unc-6*/Netrin- and *unc-40*/DCC-dependent manner. Loss of function of the genes *rib-1* or *rib-2* partially suppresses this forced dorsal migration, indicating that *unc-6*/Netrin signaling depends on functional HS chains. Null allele *unc-6(ev400)* was used in this experiment. Scale bar, 5 μm. **D.** HS modifying enzymes function in the *unc-6/*Netrin- and *slt-1/*Slit mediated-guidance of the AVM axon. Complete loss of HS modifying enzymes, including the epimerase *hse-5* and the sulfotransferases *hst-2* and *hst-6*, enhance the AVM guidance defects of *unc-6/*Netrin and *slt-1/*Slit mutants null mutants [*unc-6(ev400)* and *slt-1(eh15)*], consistent with the notion that proper HS chain modifications are important in guiding AVM through both the *unc-6/*Netrin and *slt-1/*Slit pathways, and/or possibly for another unidentified pathway. Error bars are standard error of the proportion. Asterisks denote significant difference: *** *P* ≤ 0.001, ** *P* ≤ 0.01, * *P* ≤ 0.05 (z-tests, P values were corrected by multiplying by the number of comparisons). ns, not significant. See **S6, S7, and S8 Tables**.

Prior studies have addressed roles of specific HSPG *core* proteins and HS chain *modifications* in Netrin- and Slit-mediated axon guidance in worms [8, 10–15, 18, 71], flies [72, 73], and mice [49]. HS chain *elongation per se* has been implicated in Netrin- and Slit-mediated axon guidance using *in vitro* spinal cord and retinal explant assays [74, 75]. However, an *in vivo* study of the impact of HS chain *elongation* in Netrin- and Slit-mediated guidance events has been lacking. To address how globally disrupting HS chain elongation affects guidance events that require the *unc-6*/Netrin or *slt-1*/Slit pathways, we studied the guidance of the AVM axon **(Fig 9A)**. Two complementary and highly conserved guidance pathways guide the AVM axon: attraction mediated by the UNC-40/DCC receptor towards ventral UNC-6/Netrin, and repulsion mediated by the SAX-3/Robo receptor away from dorsal SLT-1/Slit (**Fig 9B**) [68, 76–81]. Simultaneous complete loss of both *unc-6/*Netrin and *slt-1/*Slit function leads to fully penetrant AVM axon guidance defects, where ~95% of AVM axons fail to extend ventrally, as demonstrated in [77] (also reproduced by [8]). We found that *rib-1(qm32)*^m-/- z-/-^ and *rib-2(qm46)*^m-/- z-/-^ mutants are defective in AVM ventral axon guidance (**Fig 4C**), a result that is consistent with the notion that loss of HS chain elongation may affect signaling through either *unc-6/*Netrin signaling, *slt-1*/Slit signaling, or both. It is also possible that an unidentified pathway, also involving HS chains, may help guide AVM ventrally.

To test whether HS elongation contributes to both Netrin and Slit signaling pathways, we constructed double mutants of *rib-1(qm32)* and *rib-2(qm46)* with mutations in *unc-6*/Netrin and *slt-1*/Slit. Because *rib-1* and *rib-2* null alleles are lethal, we used the hypomorphic alleles *rib-1(qm32)* and *rib-2(qm46)*. For *unc-6*, we used the hypomorphic allele *e78* [82], as we found that double mutants with the null allele *unc-6(ev400)* [83] *rib-1(qm32);unc-6(ev400)* and *rib-2(qm46);unc-6(ev400)* died as embryos. For *slt-1*, we used the presumptive null allele *eh15* [77], as well as a condition of altered *slt-1*/Slit signaling, where misexpressing *slt-1*/Slit in all body wall muscles (using P*myo-3*::*slt-1*), leads to AVM axon guidance defects [84]. We found that all four double mutants (a) *rib-1(qm32)*^m-/- z-/-^*;unc-6(e78)*, (b) *rib-2(qm46)*^m-/- z-/-^*;unc-6(e78)*, (c) *rib-1(qm32)*^m-/- z-/-^*;slt-1(eh15)*, and (d) *rib-2(qm46)*^m-/- z-/-^*;slt-1(eh15)*, have AVM axon guidance defects that are more pronounced than the respective single mutants (**Fig 9B**). Similarly, animals expressing P*myo-3*::*slt-1* in the *rib-1(qm32)*^m-/- z-/-^ or *rib-2(qm46)*^m-/- z-/-^ mutant backgrounds have severe AVM guidance defects compared to the *rib-1(qm32)*^m-/- z-/-^ and *rib-2(qm46)*^m-/- z-/-^ single mutants or to animals misexpressing P*myo-3*::*slt-1* in the wild-type background (**Fig 9B**). While hypomorphic alleles complicate the interpretation of results, the finding that disruption of HS chain elongation alters axonal guidance in an additive manner to dysfunctional *unc-6/*Netrin and *slt-1*/Slit signaling, highlights the importance of HS chain elongation in axon guidance and is consistent with a role of HS chain elongation in *unc-6/*Netrin and *slt-1*/Slit signaling to guide AVM.

To directly test the functional importance of HS chain elongation in *unc-6*/Netrin signaling, we used a gain-of-function approach that specifically assays a *unc-6*/Netrin-dependent guidance event. Similar to AVM, the PVM axon is attracted ventrally towards secreted UNC-6/Netrin via the UNC-40/DCC receptor. However, misexpression of the repulsive UNC-6/Netrin receptor, UNC-5/UNC5, using the transgene P*mec-7*::*unc-5* ([85], **Fig 9C**) in PVM, results in an *unc-6*/Netrin- and *unc-40*/DCC-dependent abnormal extension of the PVM axon towards the dorsal side of the animal [86]. As controls, dorsal extension of the PVM axon is never observed in wild type or mutants in the *unc-6/*Netrin or *slt-1/*Slit signaling pathways (**Fig 9C**). We focused on the PVM axon in this assay as both the AVM and ALMR axons extend dorsally in P*mec-7*::*unc-5* transgenic animals rendering AVM indistinguishable from ALMR. We generated *rib-1(qm32)*^m-/- z-/-^ or *rib-2(qm46)*^m-/- z-/-^ mutant strains carrying the P*mec-7*::*unc-5* transgene [85] to misexpress *unc-5* in PVM. If loss of *rib-1* and *rib-2* functionally disrupts *unc-6*/Netrin signaling, we would expect to see a decrease in the *unc-5-*mediated dorsal migration of PVM. Indeed, we found that *rib-1(qm32)* and *rib-2(qm46)* loss of function significantly suppressed the *unc-6/*Netrin-dependent *unc-5*-mediated dorsal migration of PVM (**Fig 9C**, **S8 Table**), indicating that *unc-6*/Netrin signaling requires HS chain elongation. This suppression of the dorsal extension of the PVM axon by mutations in *rib-1* and *rib-2* is specific, as individual loss of function of other genes required for guidance, such as *sdn-1*/Syndecan, *slt-1*/Slit and *sax-3*/Robo, did not suppress these abnormal dorsal extensions (**Fig 9C**). Taken together our observations support the notion that HS chain elongation plays a critical role in *unc-6*/Netrin-mediated guidance. Furthermore, if *unc-6*/Netrin and *slt-1*/Slit signaling pathways were indeed the sole two key pathways guiding the AVM axon ventrally, as the fully penetrant defects of *unc-6 slt-1* double null mutants suggest [8, 77], then our results would support that HS chain elongation is important for both *unc-6*/Netrin and *slt-1*/Slit signaling. This notion is consistent with prior work implicating the HSPG syndecan in *slt-1/*Slit-mediated guidance [8, 14, 72, 73], the HSPG glypican in *unc-6/*Netrin-mediated guidance [8], and HS modifications in *slt-1*/Slit signaling [10, 11].

### HS chain modifications in *unc-6/*Netrin- and *slt-1/*Slit-mediated AVM axon guidance

Once synthesized, HS chains become extensively modified by epimerases and sulfotransferases (reviewed in [1], **Fig 3A**). In *C*. *elegans*, key modifying enzymes have been studied, including glucuronyl C5-epimerase encoded by *hse-5*, 2O-sulfotransferase encoded by *hst-2*, and 6O-sulfotransferase encoded by *hst-6* [9–11, 15, 87]. These HS modifying enzymes are required for axon guidance as mutations disrupting their function impair this process in a number of developmental contexts [1, 9–15, 21]. However, the roles of these HS modifying enzymes in the guidance of the AVM axon, which is mediated by *unc-6/*Netrin- and *slt-1/*Slit, are unknown. To determine the functional importance of specific HS modifications in AVM axon guidance, we first analyzed single, double, and triple null *hse-5*, *hst-2* and *hst-6* mutants, and found that loss of each single HS modifying enzyme led to minimal AVM axon guidance defects (**Fig 9D**), as has previously been reported [10]. However, *hse-5; hst-6* and *hst-2 hst-6* double mutants, in which the 6O-sulfotransferase and either the 2O-sulfotransferase or the C5-epimerase are mutant, display significant AVM guidance defects (**Fig 9D**). The defects of these two double mutants are not further enhanced by the loss of the third key HS modifying enzyme in *hse-5; hst-2 hst-6* triple mutants (**Fig 9D**). These observations indicate some level of compensation between HS chain modifying enzymes, which has been observed at the biochemical level [87], and suggest that combinations of types of HS chain modifications impact the guidance of AVM, which relies on *unc-6/*Netrin- and *slt-1/*Slit-signaling. Our results add to prior work showing that specific HS chain modifications regulate precise cell and axon migration events in several other contexts, including of migration events that are *slt-1/*Slit- and *unc-6/*Netrin-dependent, and through interactions with the *slt-1/*Slit signaling pathway [10–15, 18].

Next, we analyzed AVM ventral axon guidance in double mutant animals lacking just one of the HS modifying enzymes, *hse-5*, *hst-2*, and *hst-*6, and *unc-6/*Netrin or *slt-1/*Slit. We found that loss of function of any of the HS modifying enzymes enhanced the AVM guidance defects of *unc-6*/Netrin null mutants. Similarly, loss of any of the HS modifying enzymes *hse-5*, *hst-2*, or *hst-6* enhanced the AVM guidance defects of presumptive null mutants for *slt-1*/Slit [77] (**Fig 9D**). These results show that HS chain sulfations and epimerizations carried out by *hse-5*, *hst-2*, or *hst-6* enzymes participate in AVM axon guidance likely through the two key signaling pathways *unc-6/*Netrin- and *slt-1/*Slit. These findings are in agreement with prior studies that demonstrated the importance HS chain modifications to neural development in other contexts [9–15, 18, 21].

## Discussion

Animal development and tissue homeostasis rely on the regulation of molecules that instruct cellular responses. HSPGs regulate morphogens and guidance cues in the extracellular environment, but their mechanisms are still not well understood, including how multiple HSPGs function together to coordinate cellular responses. Here, we identify viable hypomorphic mutations in the genes *rib-1* and *rib-2* encoding the HS copolymerase of *C*. *elegans*. These mutations severely reduce HS levels and disrupt morphogenesis and nervous system development. We show that the coordinated action of HSPGs from various tissues contributes to guide cellular migrations during development.

### Viable hypomorphic mutations of the exostosin glycosyltransferases in *C*. *elegans*

In this study we have molecularly identified and characterized two mutations that were previously isolated in a forward genetic screen for maternally rescued uncoordinated mutants [54–56]. We show that *mum-1(qm32)* and *mum-3(qm46)* are partial loss-of-function mutations in the genes *rib-1* and *rib-2*, respectively, which encode the two exostosin glycosyltransferases that compose the HS copolymerase in *C*. *elegans*. The enzymatic activities predicted by sequence homology have been corroborated using bacterially expressed RIB-1 and RIB-2 [32, 34]. RIB-2 functions as an alpha1,4-N-acetylglucosaminyltransferase that has both GlcNAc transferase I and II activities and is involved in the addition of the first GlcNAc residue onto the tetrasaccharide linker, as well as in the elongation of HS chains [34, 57]. RIB-1 and RIB-2 glycosyltransferases together form a functional heterodimer that catalyzes HS chain elongation [32]. Moreover, HS levels have been shown to be reduced in maternally rescued worms of *rib-1(tm516)*^m+/- z-/-^ and *rib-2(qa4900)*^m+/- z-/-^ null mutations [32, 34]. Here we show that homozygous hypomorphic single mutants *rib-1(qm32)*^m-/- z-/-^ and *rib-2(qm46)*^m-/- z-/-^ have profoundly disrupted HS levels: we found that the global HS levels are severely reduced in these single mutants, and that high molecular species of LON-2/Glypican and SDN-1/Syndecan, likely corresponding to the core protein with HS chains attached, are undetectable in the *rib-1(qm32)* and *rib-2(qm46)* single mutants. These findings indicate that *rib-1(qm32)*^m-/- z-/-^ and *rib-2(qm46)*^m-/- z-/-^ result in a loss of function of the genes *rib-1* and *rib-2*. It is noteworthy that *rib-1(qm32)*^m-/- z-/-^ and *rib-2(qm46)*^m-/- z-/-^ single mutants each display severe mutant phenotypes, indicating that *rib-1* and *rib-2* cannot substitute for each other, consistent with their specific biochemical roles in HS chain elongation. In sum, we provide further evidence that the function of RIB-1 and RIB-2 is required for HS biosynthesis in *C*. *elegans*.

Several observations indicate that *rib-1(qm32)* and *rib-2(qm46)* are partial loss-of-function mutations: (a) their phenotype is less severe than deletion alleles; (b) the phenotype of *rib-1(qm32)* and *rib-2(qm46)* over a deficiency is more severe that in homozygous mutants [54]; and (c) simultaneously disrupting both genes results in complete embryonic lethality. Thus, despite the severe reduction in HS in *rib-1(qm32)*^m-/- z-/-^ and *rib-2(qm46)*^m-/- z-/-^ single mutants, residual HS copolymerase activity appears to be sufficient for viability in the single mutants. However, that disrupting both genes in *rib-1(qm32)*^m-/- z-/-^*; rib-2(qm46)*^m-/- z-/-^ leads to a more severe phenotype is likely because the HS copolymerase, a heterodimer of RIB-1 and RIB-2 proteins, is more drastically impaired in the double mutants. We propose that HS copolymerase dimers composed of one mutant protein and one wild-type protein might be stabilized by the presence of one normal protein in the complex in single *rib-1(qm32)* and *rib-2(qm46)* hypomorphic mutants (i.e. mutant RIB-1 and wild-type RIB-2 in *rib-1(qm32)*, and mutant RIB-2 and wild-type RIB-1 in *rib-2(qm46)*).

In contrast to the single mutants *rib-1(qm32)*^m+/- z-/-^ or *rib-2(qm46)*^m+/- z-/-^, which are *completely* maternally rescued [54], double mutant animals *rib-1(qm32)*^m+/- z-/-^*;rib-2(qm46)*^m+/- z-/-^ are not, and instead become severely uncoordinated and egg-laying defective adults (**Table 1**). Thus, maternal product deposited in the oocyte is sufficient to support HS copolymerase activity and allow for normal development and behavior in single hypomorphic mutants *rib-1(qm32)*^m+/- z-/-^ and *rib-2(qm46)*^m+/- z-/-^, but is insufficient for double hypomorphic mutants. Incomplete maternal rescue effect is observed for single null mutants *rib-1(tm516)*^m+/- z-/-^ and *rib-2(qa4900)*^m+/- z-/-^ [32, 34]. High levels of *rib-1* and *rib-2* transcripts are detected in the germline of *C*. *elegans* (http://nematode.lab.nig.ac.jp/). These observations highlight the importance of HS copolymerase activity, and therefore HSPGs, from the earliest stages of development.

RIB-1 and RIB-2 are not expected to affect the biosynthesis of glycosaminoglycans other than HS. In *C*. *elegans*, both HS and CS, but not hyaluronate nor dermatan sulfate, have been detected [88, 89]. Whereas HS and CS chains share the same tetrasaccharide linker to couple the HS or CS chain to their respective core proteins, the elongation of HS and CS chains are carried out by different enzymes. The elongation of CS chains, a polymer of alternating GlcA and N-acetylgalactosamine (GalNAc) residues, is catalyzed by a bifunctional glycosyltransferase encoded by the *sqv-5* gene [90]. Thus, *rib-1(qm32)* and *rib-2(qm46)* mutations facilitate the study of the consequences of globally and specifically disrupting HS elongation in live animals.

### HS elongation is required for morphogenesis and cell migrations

Complete disruption of HS chain elongation in the deletion alleles of *rib-1(tm516)*^m-/- z-/-^ and *rib-2(qa4900)*^m-/- z-/-^ affects the mutant organism in a pleiotropic fashion, leading to fully penetrant embryonic lethality [32, 34], which had limited the systematic study of the impact of HS chain elongation in later developmental processes. We found that a basal level of the required enzymatic activities in the hypomorphic mutants *rib-1(qm32)*^m-/- z-/-^ and *rib-2(qm46)*^m-/- z-/-^ is sufficient to bypass major pleiotropic effects, allowing a proportion of the animals to fully develop and reach adulthood [54]. In these animals that complete development, major morphogenetic movements, such as gastrulation, ventral closure and organogenesis, occurred normally, and their anatomy, including the specification of neuronal identities and the layout of ganglia and major axon fascicles, was grossly normal. This hypomorphic condition allowed us to study the influence of HS chain elongation on the guidance of cell and axon migration in viable animals. We found that disrupting HS chain elongation affects the migrations of neurons and axons, including migrations that occur during embryonic and post-embryonic development, and along both body axes (antero-posterior and dorso-ventral) [78]. It is worth noting that the motility *per se* of migrating cells is not lost in *rib-1(qm32)*^m-/- z-/-^ and *rib-2(qm46)*^m-/- z-/-^ mutants as soma and axons often overshoot their targets. Rather, it is the guidance of migrations during development that is disrupted by the loss of function of *rib-1* and *rib-2*. The critical role of HS elongation in axon guidance during nervous system development is evolutionarily conserved, as disruption of HS elongation in mice and fish leads to defective axonal guidance [40, 49].

Previous analyses of the consequences of disrupting HS chain elongation on neuronal development reported weaker defects than those we describe here using *rib-1(qm32)*^m-/- z-/-^ and *rib-2(qm46)*^m-/- z-/-^. This is not surprising since only maternally rescued animals *rib-1(tm516)*^m+/- z-/-^ and *rib-2(tm710)*^m+/- z-/-^, or partial knock down of gene activities with *rib-1(RNAi)* and *rib-2(RNAi)*, could be studied before. For example, HSN soma migration is fully normal in maternally rescued *rib-2(tm710)*^m+/- z-/-^ [21] and 27% of maternally rescued *rib-1(tm516)*^m+/- z-/-^ animals exhibit HSN axon guidance defects [32]. Similarly, *rib-1(RNAi)* and *rib-2(RNAi)* lead to a 40–45% penetrance of combined HSN cell and axon guidance events [17]. In contrast, 77% and 74% of *rib-1(qm32)*^m-/- z-/-^ and *rib-2(qm46)*^m-/- z-/-^ mutant animals exhibit abnormal HSN soma migration, and 100% and 83% of *rib-1(qm32)*^m-/- z-/-^ and *rib-2(qm46)*^m-/- z-/-^ mutant animals display defects in the guidance of the HSN axon, respectively. This highlights that the newly identified hypomorphic *rib-1(qm32)* and *rib-2(qm46)* mutants reported here enable the study of HS chain elongation-dependent biological processes.

The guidance of multiple migrating neurons and axons is altered in *rib-1(qm32)*^m-/- z-/-^ and *rib-2(qm46)*^m-/- z-/-^ mutants, suggesting that disruption of HS chain elongation impacts several guidance pathways. In particular, our analysis of the ventral axon guidance of AVM shows that HS chain elongation and HS chain modifications, are important for signaling via the *unc-6*/Netrin and the *slt-1*/Slit signaling pathways, and perhaps a yet to be identified HS-dependent pathway. That loss of function of *rib-1* or *rib-2* was able to suppress the *unc-6/*Netrin-dependent dorsalization of PVM upon ectopic expression of *unc-5/*UNC5 supports the model that *unc-6*/Netrin signaling requires HS chains. We have previously shown that HSPG *lon-2/*Glypican functions with, and is required for, *unc-6/*Netrin signaling in axon guidance [8]. Interestingly, the core protein of LON-2/glypican, but not its HS chains, functions in the *unc-6*/Netrin pathway. In fact, two versions of LON-2/Glypican lacking the HS chains are able to function in *unc-6/*Netrin-mediated axon guidance (one where the HS attachment sites were deleted [67], which indeed prevents the addition of HS onto LON-2/Glypican (**Fig 3C**), and one where LON-2/Glypican is truncated in a way that removes all HS attachment sites, [8]). Taken together, the observation that the core protein of LON-2/Glypican, but not its HS chains, functions in *unc-6/*Netrin signaling, and that HS elongation is required for *unc-6*/Netrin signaling (loss of *rib-1* or *rib-2* suppresses the unc-6/Netrin-dependent effect of *unc-5*/UNC5 ectopic expression), raises the possibility that an additional unidentified HSPG functions in *unc-6/*Netrin signaling. One HSPG that has a role in *unc-6*/Netrin guidance in other contexts is *unc-52*/Perlecan, which affects the guidance timing defects of distal tip cells upon ectopic early expression of *unc-5/*UNC5 [16]. However, loss of *unc-52* alone does not affect AVM axon guidance and does not enhance defects of *sdn-1/*Syndecan mutants [8], indicating that *unc-52* likely does not participate in AVM guidance. Thus, another unidentified HSPG may function in *unc-6/*Netrin signaling through its HS chains.

**Fig 10:**
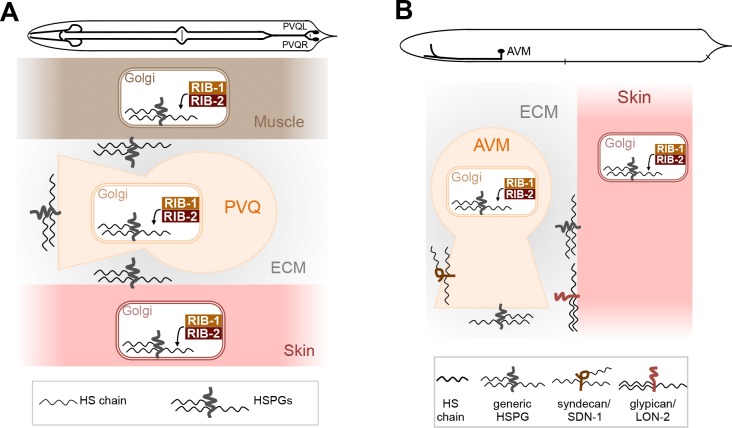
A model for the role of HSPGs in the guidance of PVQ and AVM axons. **A.** The PVQ axons extend along the ventral nerve cord, following pioneer axons [96]. PVQ axons are in contact with the extracellular matrix and in close proximity to muscles and hypodermis (skin). For proper guidance of the PVQ axons, combined HS chain elongation from the muscles, skin, and neurons is required, suggesting that multiple HSPGs coordinate the guidance of the PVQ axon. **B.** The ventral axon guidance of AVM requires HS chain elongation from the migrating neuron itself, or from the skin, and HS chains are required for both *unc-6*/Netrin and *slt-1*/Slit guidance systems. Taken together with previous results, AVM is likely guided by HSPGs functioning from AVM (including *sdn-1/*Syndecan [8]), and HSPGs functioning from the skin, (including *lon-2/*Glypican [8]), as well as possibly other unidentified HSPGs.

Also, the notion that HS chains are important for *slt-1*/Slit signaling is consistent with prior reports that (1) HS chain elongation is required for retinal explant axon outgrowth *in vitro* [74], (2) the HSPG gene *sdn-1*/Syndecan functions in *slt-1*/Slit signaling to guide the AVM axon [8] (**Fig 10B**) and other axons (PVQ, [14]), and (3) HSPG Syndecan is key to Slit signaling and distribution in flies [72, 73]. Furthermore, studies have demonstrated roles for HS chains in *slt-1*/Slit-mediated guidance in other contexts [10, 11], through the study of HS modifying enzymes mutants, however whether it is the modifications of the HS chains on SDN-1/Syndecan specifically that are required for guidance is not known.

### HS chain elongation in multiple cell types coordinates cell migration

We analyzed the spatial requirements for the HS copolymerase by focusing on two specific migrating neurons, namely the embryonically migrating PVQ axon and the AVM axon that extends during the first larval stage. In both cases we found that *rib-1* expression in several cell types restored function during development of *rib-1* mutants. For the guidance of the migrating PVQ axon, combined HS copolymerase expression in the hypodermis, neurons, and body wall muscles of *rib-1* mutants was required to rescue PVQ axon guidance defects. This observation indicates that HS chains synthesized onto HSPGs from multiple tissue types cooperate to properly pattern the ventral midline and suggests that distinct HSPGs from specific tissues may contribute to properly guide the PVQ axons at the ventral midline (**Fig 10A**). Interestingly, the combined loss of two HSPGs, a glypican and a syndecan, in the *lon-2 sdn-1* double mutant leads to a penetrance of defects in PVQ guidance similar to *rib-1(qm32)*^m-/- z-/-^ and *rib-2(qm46)*^m-/- z-/-^ mutants [12], suggesting that PVQ axon guidance defects observed in *rib-1(qm32)*^m-/- z-/-^ and *rib-2(qm46)*^m-/- z-/-^ mutants may reflect a disruption of HS chains onto LON-2/Glypican in the hypodermis and SDN-1/Syndecan in the PVQ neurons. Consistent with this interpretation, *lon-2*/Glypican has been found to function non-cell autonomously in the hypodermis to guide the axon of AVM [8], and *sdn-1*/Syndecan has been shown to function cell autonomously in the migrating neuron in a variety of contexts, such as AVM axon guidance [8], PVQ axons, HSN soma, and ALM soma [14]. Furthermore, PVQ axon guidance likely requires that the specific HS chains on core HSPGs not only be synthesized but also modified, as the combined loss of HS modifying enzymes also leads to PVQ axon guidance defects [10]. Indeed, loss of the C5-epimerase *hse-5* and the 6-O-sulfotransferase *hst-6* in *hse-5; hst-6* double mutants, or loss of the 2-O-sulfotransferase *hst-2* in double mutants *hst-2 hst-6* leads to PVQ axon guidance defects [10] with a similar penetrance to that of *rib-1(qm32)*^m-/- z-/-^ and *rib-2(qm46)*^m-/- z-/-^ mutants. Together, our studies within the context of the literature suggest that the coordinated action of specific HS chains synthesized and modified in different tissues onto distinct HSPG core proteins function to properly guide the PVQ axons at the ventral midline.

Similarly, the defective guidance of the axon of the AVM neuron is strongly rescued by expression of the HS copolymerase in the AVM neuron itself, but expression in the underlying hypodermis also contributes to normal AVM development. In this case too, HS chains synthesized onto core HSPGs functioning to guide AVM may be *sdn-1*/Syndecan in AVM and *lon-2*/Glypican in the hypodermis, as previously identified by analysis of core protein mutants in the context of AVM axon guidance [8] (**Fig 10B**).

### HSPGs are expressed in multiple cell types throughout development

Given that HSPGs decorate most cells in metazoans and are implicated in numerous cellular processes at the cell surface, including cell-matrix, cell-cell, and ligand-receptor interactions during development and tissue homeostasis, it could be expected that the HS copolymerase may be expressed ubiquitously. Functional *rib-1*::*gfp* was detected in virtually all cell types at some point during development. Interestingly, the HS copolymerase expression pattern was found to be dynamic, with levels changing and differing across tissues and developmental stages. Expression is strong and transient in hypodermal cells of the embryo, the larva, and the developing vulva, likely reflecting the developmental requirements for cell migration during the formation of complex tissue shapes during morphogenesis. Our observations support a model in which high expression of the HS copolymerase in cells that secrete a basement membrane, such as hypodermal cells, is pivotal for cell migration along this basement membrane. In addition, it is quite likely that migrating cells themselves might dynamically regulate surface HSPGs to modulate their adhesion properties and regulate the signaling of guidance cues. This is reflected by HS copolymerase expression peaking during periods of cell migration during embryogenesis and vulva formation.

In addition to this dynamic expression pattern, the HS copolymerase shows sustained expression in a number of structures throughout the life of the animal, including the pharynx, the pharyngeal-intestinal valve, the anal depressor, sphincter, and enteric muscles, as well as the nervous system. These are morphologically complex cells that are under continuous mechanical stress. For instance, the pharynx is constantly pumping bacteria, thus exerting variable pressure on the pharynx itself and the pharyngeal-intestinal valve. Similarly, the enteric muscles, the anal depressor, and the sphincter, all contract to expel waste, and, as the animal moves, the relatively long neuronal axons within the nerve cords are constantly subjected to stretch and relaxation. Other cell types expressing the HS copolymerase are the spermatheca, which stretches to welcome oocytes to be fertilized and contracts to expel the zygotes, and the uterine muscles, which contract to lay embryos in reproducing adults. Our observations point to a role for HSPGs in maintaining the integrity of tissues, possibly by regulating the attachment of cells that undergo considerable mechanical stress from repeated body contractions, and thus contribute to tissue homeostasis. That *rib-1* expression persists post-developmentally is consistent with studies in other model systems that describe post-developmental roles for HS and HSPGs [4, 50].

The *rib-1* expression pattern overlaps with known expression patterns of specific HSPG core proteins. For example, membrane bound SDN-1/syndecan is expressed in neurons, hypodermis, and pharynx [14], GPI-linked LON-2/glypican shows expression in the intestine and hypodermis [66], and UNC-52/perlecan, a secreted HSPG, is expressed in body wall muscles, digestive system muscles, and pharynx [91, 92]. Overlap between expression of HS biosynthetic machinery, such as RIB-1, and the localization of specific HSPGs, suggests that HSPGs may remain near cells where HS synthesis occurs. This may have functional relevance, as glypicans in fibroblast cells were shown to be internalized through endocytosis, returned to the Golgi, and then transported back to the membrane with HS chains altered both in length and modification pattern [93, 94]. Whether an internal recycling of HSPGs back to the HS biosynthetic machinery in the Golgi occurs in *C*. *elegans*, or whether it has functional relevance to guidance, remains to be determined.

In conclusion, our studies have identified viable mutations in each of the two subunits of the HS copolymerase in *C*. *elegans*, which severely disrupt HS biosynthesis, leading to profound developmental defects. Our findings offer a model system to dissect the functions of HSPGs in *C*. *elegans* and uncover general principles of their roles during development and tissue homeostasis.

## Materials and Methods

### Nematode strains and genetics

Nematode cultures were maintained at 20°C on NGM plates seeded with OP50 bacteria as described [51]. *mum-1/rib-1(qm32)* and *mum-3/rib-2(qm46)* alleles were outcrossed five times before building strains with reporters. 14% of *mum-1/rib-1(qm32)*^m-/- z-/-^ animals reach adulthood, as 68% of the embryos hatch into larvae and 20% of larvae reach adulthood; and 63% of *mum-3/rib-2(qm46)*^m-/- z-/-^ animals reach adulthood, as 85% of the embryos hatch into larvae, and 74% of larvae reach adulthood [54]. Alleles used in this study are listed in **S1 Table**. Strains were constructed using standard genetic procedures and are listed in **S9 Table**. When needed, genotypes were confirmed by genotyping PCR or sequencing, using primers listed in **S10 Table**.

### Neuroanatomical observations

Neuroanatomy was examined in animals of *rib-1(qm32)*^m-/- z-/-^ and *rib-2(qm46)*^m-/- z-/-^ that are had completed development to L4 larvae and adults (14% and 63% of the respective populations) using specific reporters. Animals were mounted on agarose pads, anaesthetized with 100 mM sodium azide, and examined under a Zeiss Axio Scope.A1 or a Zeiss Axioskop 2 Plus.

#### CAN soma and axon

CAN was examined using the reporter *lqIs4*. Axon guidance was examined only in animals with properly migrated CAN cell (as when the cell body is abnormally positioned, the axon may no longer be in contact with the appropriate guidance systems). Axons were scored as defective when they were located away from the lateral aspect of the animal, either ventrally or dorsally, when they failed to extend fully, or exhibited branching. Cell bodies were scored as defective for migration when they were located either at or anterior to the midpoint between the pharynx and the vulva (the wild-type CAN soma position is near the vulva). CAN soma was virtually never found posterior to the vulva.

#### PVQ axon

PVQ was examined using the reporter *hdIs29*. Animals were scored as defective when axons projected laterally, fasciculated inappropriately, or failed to extend fully.

#### AVM axon and soma

AVM neurons were examined in L4 larvae and adult animals using the reporter *zdIs5*. Worms were counted as having an AVM soma migration defect when AVM was located posterior to the vulva. Animals with the cell body of AVM posterior to the vulva (cell migration defect) were excluded from axon guidance analysis, but included in **Figs 1 and 2C** for % Defective soma and axon. Worms were counted as mutant for AVM ventral axon guidance if: **a)** AVM failed to send an axon ventrally and instead projected laterally to the anterior; or **b)** the AVM axon projected laterally, in the anterior or the posterior direction, for at least ~15 μm (more than three AVM cell body diameters) before projecting to the ventral side. When the angle between the initial anterior/posterior axon projection and the ventral axon projection was >45°, it was counted as mutant; thus, AVM axons with a slight curve in their ventral trajectory were not considered defective.

#### PVM axon

Axons of neurons PVM were examined in L4 larvae and adult animals using the reporter *zdIs5*. Worms were counted as having their PVM axon misoriented dorsally if the axon of PVM was projecting to the dorsal side of the animal. The vulva was used as a reference for the ventral side. Worms carrying the transgene *evIs25* (P*mec-7*::*unc-5*) whose *zdIs5* labeled neurons were too misplaced to be reliably identified were excluded from the analysis.

#### HSN soma and axon

HSN was examined using the reporter *zdIs13* in young adult animals. Animals were scored as defective for HSN axon guidance when axons projected laterally, posteriorly, fasciculated inappropriately, or failed to extend fully. Axon guidance was examined only in animals with properly migrated HSN cell (as when the cell body is abnormally positioned, the axon may no longer be exposed to the appropriate guidance systems). Cell bodies were scored as defective for migration when they were either anterior to the vulva, or either at or posterior to the midpoint between the vulva and the tail.

#### Motorneuron commissures

Cholinergic and GABAergic motoraxons were examined using the reporters *vsIs48* P*unc-17*::*gfp* and *ufIs34* P*unc-47*::*mCherry*, respectively. Commissures contain one or two axons, and only commissures located between the terminal bulb of the pharynx (in the head) and the anal depressor (in the tail of the animal) were examined.

#### Cholinergic

In wild-type animals, most cholinergic motoraxons extend along the right side of the animal, such that only seven cholinergic motoraxons (of motorneurons DA3, DB4, DA4, DB5, DA5, DA6, DA7) extend on the left side of the animal. Animals were scored as defective when more than the normal set of seven cholinergic motorneuron commissures were present on the left side of the animal, similar to quantification described previously [10]. Having extra motoraxons on the left side of the animal indicates that these axons were misguided at the ventral midline, abnormally extending along the left side instead of along the right side of the animal.

#### GABAergic

In wild-type animals, most GABAergic motoraxons are located on the right side of the animal, such that only two GABAergic commissures are located on the left side of the animal (of motorneurons DD1 and VD2). Animals were scored as defective when more than two GABAergic commissures were present on the left side of the animal, similar to quantification described previously [10].

#### Dorsal cord

The dorsal nerve cord was examined using the reporter *evIs111*. Animals were scored as having a defasciculated dorsal nerve cord when the cord was split into two or more bundles for any length of the dorsal nerve cord, instead of running as a single tight bundle as seen in the wild type.

#### Excretory canals

Excretory canals were examined using reporter *bgIs312*. Wild-type animals have four lateral canals, two projecting anteriorly in the head and two projecting posteriorly along the length of the body. Animals were scored as defective when a canal was located in an abnormal position (including when an anterior canal extended posteriorly, or a posterior canal extended anteriorly, or a canal laid ventrally or dorsally instead of laterally), when canals were too short (i.e. a posterior canal did not extend beyond the vulva), or when an abnormal number of canals was present.

#### DTC guidance analysis

The path of migration of the DTC brings about the shape of the mature gonad arms. In the wild type, the DTC migrates away from the vulva location along the antero-posterior axis of the animal. The DTC then turns dorsally to reach the dorsal side of the animal. Once the DTC reaches the dorsal aspect, it migrates back towards the vulva along the antero-posterior axis of the animal. To infer the path of DTC migration, gonad arms were examined in late L4 and young adult animals using DIC microscopy. Animals were counted as having abnormal gonad arms when a) the anterior gonad arm was on the left side instead of being on the right side; b) the posterior gonad arm was located on the right side instead of the left side; c) the distal portion of a gonad arm was located ventrally instead of dorsally; d) the proximal portion of the gonad arm was located dorsally instead of ventrally; or e) the proximal gonad arm was too short, resulting from a premature turn of the DTC towards the dorsal side.

#### Excretory glands

Excretory glands were examined in larvae using DIC microscopy. Animals were counted as having abnormally located excretory glands when they were anterior of the pharyngeal terminal bulb (excretory glands are located just posterior of the pharyngeal terminal bulb in wild-type animals).

### Genetic mapping of *mum-1/rib-1*

For mapping *mum-1*, a three-point mapping experiment was carried out by picking Unc-non-Dpy and Dpy-non-Unc recombinants from heterozygous mothers of the genotype *mum-1/unc-24 dpy-20*, and the presence of *mum-1* was assessed among the progeny of the homozygosed recombinants. Two-point mapping was carried out by picking Dpy worms from *mum-1 dpy-20/++* heterozygous mothers, and the presence of *mum-1* was assessed in the next generation. Also, Lin-non-Dpy recombinants were picked from heterozygous *mum-1/lin-3 dpy-20*.

### Microinjections and transgenic animals

As the *rib-1 and rib-2* mutants are severely morphologically abnormal, cosmids, constructs, and PCR products were injected into strains carrying the *mum-1/rib-1(qm32)* or *mum-3/rib-2(qm46)* mutations in a heterozygous state, balanced by flanking visible markers. For *rib-1*, we used *rib-1(qm32)/unc-24(e138) dpy-20(e1282ts)* and for *rib-2*, we used *rib-2(qm46)/unc-32(e189) dpy-18(e364)* (see **S9 Table**). Transgenic F1s were isolated and lines homozygous for *rib-1* or *rib-2* were established.

Transgenic animals were generated by standard microinjection techniques [95]. Each construct or PCR amplicon was injected at 5 to 25 ng/μl with one or two coinjection markers which included pRF4-*rol-6(su1006d)* (100–150 ng/μL), P*ttx-3*::*mCherry* (50 ng/μL), P*ceh-22*::*gfp* (50 ng/μL), pCB101.1 P*rgef-1*::*DsRed2* (50 ng/μL), and P*unc-122*::*rfp* (50 ng/μL). pBSK+ (90–100 ng/μL) used to increase total DNA concentration if needed. For coinjection markers used for each rescued transgenic line, see **S9 Table**.

### Rescue and expression constructs

The gene *rib-1*/F12F6.3 is downstream of the gene *srgp-1*/F12F6.5 in an operon of two genes. The nearest gene upstream of the operon is transcribed in the opposite direction. The genomic region between the operon of *srgp-1* and *rib-1*, and the upstream neighboring gene is 4352 bp, corresponding to coordinates 22290–26642 on cosmid F12F6.

**P*rib-1*::*rib-1* (PCR product):** A PCR product containing bases 34593–39595 of cosmid F12F6 of the *rib-1* locus was amplified with *Pfu* polymerase.

**P*rib-2*::*rib-2* (PCR product):** A PCR product containing bases 581 to 6196 of cosmid K01G5 of the *rib-2* locus was amplified with Phusion polymerase.

**P*rib-1*::*gfp* (pCB78):** A PCR generated piece containing bases 23701 to 26662 of cosmid F12F6 corresponding to the promoter region of the *rib-1* operon, as well as the initial 7 codons of *srgp-1*, was cloned upstream of *gfp* in the pPD95.77 vector using enzymes *Pst*I and *Xba*I.

**P*rib-1*::*rib-1*::*Venus* (pCB221):** The *rib-1* promoter region containing bases 23701–26580 of cosmid F12F6 was PCR amplified and cloned upstream of *gfp* in the pPD95.77 vector using enzymes *Sph*I and *Pst*I. A PCR generated piece containing bases 34452–39527 of cosmid F12F6 corresponding to the intergenic sequence between the genes *rib-1* and *srgp-1*, as well as the *rib-1* coding sequence, was cloned downstream of the *rib-1* promoter and upstream of *gfp* using enzymes *Pst*I and *Avr*II. Then, *gfp* was replaced with a PCR amplified *Venus* and cloned in frame with *rib-1* using enzymes *Msc*I and *Apa*I.

As a note, for *rib-*2, we constructed several transcriptional P*rib-2*::*gfp* and translational P*rib-2*::RIB-2::Venus reporters with different sizes of promoter region, injected at a range of concentrations (10–150 ng/μL). At least three transgenic lines were examined for each condition, but gave no or a very weak expression level in transgenic worms carrying these constructs. A very faint level of P*rib-2*::*gfp* was broadly detected in comma-stage embryos, and in the head and vulva area at later developmental stages.

**P*dpy-7*::*rib-1* cDNA (pCB186):** The *rib-1* cDNA was amplified from yk1228g12 and ligated into a P*dpy-7* vector with a pPD95.75 backbone using enzymes *Xma*I–*Nco*I.

**P*myo-3*::*rib-1* cDNA (pCB196):** A P*myo-3 Hind*III–*Xba*I fragment was ligated upstream of the *rib-1* cDNA in a pCB186 *Hind*III–*Xba*I fragment in place of P*dpy-7*.

**P*mec-7*::*rib-1* cDNA (pCB204):** The *rib-1* cDNA was amplified from yk1228g12 and cloned into the pPD96.41 vector using enzymes *Age*I–*Bgl*II.

**P*rib-1*::*rib-1* cDNA (pCB225):** The *rib-1* cDNA was ligated downstream of the *rib-1* promoter (bases 23,701 to 26,580 of cosmid F12F6) using enzymes *Xma*I–*Apa*I in the pPD95.77 backbone.

**P*rgef-1*::*rib-1* cDNA (pCB199):** The *rib-1* cDNA was ligated downstream of P*rgef-1* in place of DsRed2 using enzymes *Xma*I–*Apa*I in the pCB101.1 vector.

All inserts of finalized clones were verified by sequencing.

### Molecular analysis of *mum-1/rib-1* and *mum-3/rib-2* mutant alleles

The genomic regions of *mum-1/rib-1* and *mum-3/rib-2* were PCR amplified using *Pfu* polymerase and sequenced on two independent PCR products amplified from genomic DNA of *qm32* and *qm46*, respectively, using primers to cover the entire genomic region. Primers listed in **S10 Table** sequence over the mutation in each of the two mutants.

### Western blot analysis of SDN-1::GFP expressed in worms

Mixed-stage wild type (N2), SDN-1::GFP (*opIs171*), *rib-1*; SDN::GFP (*rib-1; opIs171*) and *rib-1* GFP control (*rib-1; lqIs4*) worms were collected from plates devoid of bacteria in buffer and protease inhibitors (Roche). Worm pellets were subjected to repeated freeze-thaw cycles. Protein concentration was measured using the Pierce 660 nm Protein Assay on a Nanodrop. 80 μg of samples mixed with 2x Laemmli sample buffer (Bio-Rad) were frozen in liquid nitrogen, then boiled, separated by SDS-PAGE on a 4–20% Mini-Protean TGX gel (Bio-Rad), and transferred to PVDF membrane. Membranes were incubated in 1:3000 rabbit anti-GFP primary antibody (Millipore #AB3080) and 1:9000 goat anti-rabbit HRP secondary antibody (Bio-Rad #166-2408EDU). For the loading control, membranes were incubated in 1:5000 rabbit anti-HSP90 antibody (CST #4874) and 1:10000 goat anti-rabbit HRP secondary antibody (Bio-Rad #166-2408EDU). Signal was revealed using Clarity Western ECL Substrate (Bio-Rad), and imaged using film (LabScientific).

### Western blot analysis of LON-2::GFP and LON-2ΔGAG::GFP expressed in worms

Mixed-stage wild type (N2), GFP control (*lqIs4*), LON-2::GFP (TLG257), LON-2ΔGAG::GFP (TLG199), *rib-1;* LON-2::GFP (VQ525), *rib-2;* LON-2::GFP (VQ528), *rib-1* GFP control (*rib-1; lqIs4*) and *rib-2* GFP control (*rib-2; lqIs4)* worms were collected from plates devoid of bacteria in buffer and protease inhibitors (Roche), mixed with 2x Laemmli sample buffer (Bio-Rad), and frozen in liquid nitrogen. Samples were boiled and spun down, separated by SDS-PAGE on a 4–20% Mini-Protean TGX gel (Bio-Rad), and transferred to PVDF membrane. Membranes were incubated in 1:3000 rabbit anti-GFP primary antibody (Millipore #AB3080) and 1:9000 goat anti-rabbit HRP secondary antibody (Bio-Rad #166-2408EDU). For the loading control, membranes were incubated in 1:5000 rabbit anti-HSP90 antibody (CST #4874) and 1:10000 goat anti-rabbit HRP secondary antibody (Bio-Rad #166-2408EDU). Signal was revealed using Clarity Western ECL Substrate (Bio-Rad), and imaged using film (LabScientific).

### Analysis of HS levels by western blot in worms

#### Protein extraction

Worms were allowed to grow for ~5 generations on ten 9 cm plates. Because these HS level assays require large pellets of thousands of worms, rather than picking worms one-by-one, we collected worms by washing populations on plates that had grown for several generations. We estimate that only ~10–20% of animals actually carried the extrachromosomal transgene P*rib-1*::*rib-1(+)* or P*rib-2*::*rib-1(+)*, respectively (as the unstable non-integrated extrachromosomal arrays is lost during cell divisions and over generations), by the time that the worms were collected from plates. Mixed-stage worms from plates devoid of bacteria were collected in 50 mM Tris buffer and protease inhibitors (Roche # 11697498001), and frozen in liquid nitrogen. Worm pellets were subjected to repeated freeze-thaw cycles and protein concentration was measured using the 660 nm Protein Assay (Pierce # 22660) on a Nanodrop.

#### Detection of HS

80 μg of protein suspended in buffer and protease inhibitors (Roche) were used per sample. Undigested controls were treated exactly like the digested samples, except for the heparinase I and III enzyme treatment. To the digested samples, 10 mU of heparinase I and III enzyme (Sigma-Aldrich # H3917) was added. All samples were incubated at 37°C for 3 hours. 2x Laemmli sample buffer (Bio-Rad) was added and samples were frozen in liquid nitrogen. Samples were boiled, separated by SDS-PAGE on a 4–20% Mini-Protean TGX gel (Bio-Rad), and transferred to PVDF membrane. Membranes were incubated in 1:200 mouse anti-Δ-Heparan Sulfate (3G10 epitope) primary antibody (Amsbio #370260–1) and 1:10000 horse anti-mouse HRP secondary antibody (Vector Labs #PI-2000). For the loading control, membranes were incubated in 1:5000 rabbit anti-HSP90 antibody (CST #4874) and 1:9000 goat anti-rabbit HRP secondary antibody (Bio-Rad #166-2408EDU). Signal was revealed using Clarity Western ECL Substrate (Bio-Rad), and imaged using film (LabScientific).

### RT-PCR analysis

Worm RNA was extracted using Trizol (Invitrogen) according to manufacturer’s instructions. RNA (500 ng) was reverse transcribed using the High Capacity cDNA Reverse Transcription Kit (Applied Biosystems) and random primers. PCR reactions were carried out with cDNA template, and 0.25 μM of each primer in 10 mM Tris pH 8.3, 1.5 mM MgCl2, 50 mM KCl, 0.2 mM deoxynucleotides, and 1 U Phusion DNA polymerase for 30 cycles of 94°C for 10 seconds, 55°C for 20 seconds, and 72°C for 45 seconds. Primers used to detect *rib-1* transcript: oCB1533 (TGGAATCGACACAACGGATCG), oCB1534 (CAAGCAGTTCGTCGTATTCCC), oCB1535 (GAATACGACGAACTGCTTGCC), oCB1536 (TCCAGCTCAATCTTGTTGTCG) and oCB1537 (AGATGTGATGAGGGGAGAACG). Primers used to detect *rib-2* transcript: oCB1538 (CAGTTCGTTTGGAATTGACGG), oCB1539 (CTGCTATATGATTGACATCCACAGG), oCB1540 (CACGTCATCACGCCAGATACG), and oCB1541 (TGATTCTGTGGGAGACGCGTC). The transcript for Y45F10D.4 was used as control using the primers oCB992 (TCGCTTCAAATCAGTTCAGC) and oCB993 (GCGAGCATTGAACAGTGAAG).

## Supporting Information

S1 Data Excel file containing, in separate sheets, the underlying numerical data for the graphs in Figs 1C, 2C, 4A, 4B, 4C, 4D, 5A, 5B, 6A, 6B, 6C, 8A, 8B, 9B, 9C and 9D. (XLSX)

S1 Table List of mutant alleles used. (DOCX)

S2 Table AVM soma and axon guidance defects in *rib-1* and *rib-2* mutants and in transgenic lines used to rescue with the respective genomic locus. (DOCX)

S3 Table PVQ guidance defects in *rib-1* and *rib-2* mutants and in transgenic lines used to rescue with the respective genomic locus. (DOCX)

S4 Table PVQ guidance defects in *rib-1* mutants and in transgenic lines for tissue-specific rescue assays. (DOCX)

S5 Table AVM axon guidance defects in *rib-1* mutants and in transgenic lines for tissue-specific rescue assays. (DOCX)

S6 Table AVM axon guidance defects in *rib-1* and *rib-2* mutants in combination with AVM guidance pathway mutants. (DOCX)

S7 Table PVM dorsal guidance defects quantified in wild-type and mutant strains, with or without misexpression of *unc-5* in the PVM neuron, using transgene *evIs25* P*mec-7::unc-5*. (DOCX)

S8 Table AVM axon guidance defects in HS-modifying enzyme mutants in combination with AVM guidance pathway mutants. (DOCX)

S9 Table List of strains used. (DOCX)

S10 Table List of primers used for building strains. (DOCX)

S1 Fig The mRNA levels for *rib-1 and rib-2* are unaffected in *rib-1(qm32)*^m-/-z-/-^ and *rib-2(qm46)*^m-/-z-/-^ mutants. **A.** Schematic of the gene structure of *rib-1* and *rib-2* (boxes are exons and lines are introns) and the primers used for RT-PCR analysis. **B.** RT-PCR performed on wild type, *rib-1(qm32)*^m-/-z-/-^ and *rib-2(qm46)*^m-/-z-/-^ mutants. *rib-1* and *rib-2* transcript levels are comparable in the wild type and *rib-1(qm32)*^m-/-z-/-^ and *rib-2(qm46)*^m-/-z-/-^ mutants.
(TIF)

S2 Fig SDN-1::GFP expression is similar in wild-type and *rib-1(qm32* ^m-/-z-/-^ mutant animals. **A.** Ventral view of mid body region of fourth larval stage animals; the asterisk denotes the vulva, and the arrows indicate the ventral nerve cord. **B**. Lateral view of fourth larval stage animals showing lateral hypodermal cells (seam cells). Analysis of SDN-1::GFP in *rib-2(qm46)*^m-/- z-/-^ mutants was not possible as a strain of *rib-2(qm46)* carrying *opIs171* [SDN-1::GFP] could not be built.
(TIF)
