# Supplementary material for: Functional Requirements for Heparan Sulfate Biosynthesis in Morphogenesis and Nervous System Development in C. elegans
Source: PLoS Genet. 2017 Jan 9;13(1):e1006525. doi: 10.1371/journal.pgen.1006525 (PMC5221758; doi:10.1371/journal.pgen.1006525)
Supplement: S1 Table — (DOCX) [file pgen.1006525.s002.docx]

**S1 Table**. List of mutant alleles used.

| **Gene** | **Allele** | **Nature of allele** | **Reference** |
| --- | --- | --- | --- |
| ***rib-1*** | *qm32* | Stop codon converted into a Lys codon, likely extending the open reading frame into the 3’UTR | This study |
| ***rib-2*** | *qm46* | Arg to Gln amino acid substitution at residue 434 | This study |
| ***hse-5*** | *tm472* | 1249 bp deletion and addition of an adenosine, which deletes most of exons 4 – 7 and generates a frame shift after exon 4 | (1) |
| ***hst-2*** | *ok595* | 1336 bp deletion, deleting from exon 4 to part of exon 7 | (1) |
| ***hst-6*** | *ok273* | 1064 bp deletion, with the addition of nucleotides CTTT, which deletes exons 4 and 5 and generates a frameshift after exon 3 | (1) |
| ***unc-6*** | *ev400* | Early stop Q78*. Null. | (2) |
| ***unc-6*** | *e78* | C410Y. Partial loss of function. | (3) |
| ***slt-1*** | *eh15* | Duplication and deletions. First copy contains a 1900 bp deletion. Both duplicated copies have a 100 bp deletion. First copy produces no mRNA while second copy produces mRNA with a frameshift. | (4) |
| ***unc-40*** | *e271* | Early stop R824*. Null. | (5) |
| ***sax-3*** | *ky123* | Deletion of signal peptide and first exon. | (6) |
| ***sdn-1*** | *zh20* | 1258 bp deletion. | (7) |

1. Bülow HE, Hobert O. Differential sulfations and epimerization define heparan sulfate specificity in nervous system development. Neuron. 2004;41(5):723-36.

2. Wadsworth WG, Bhatt H, Hedgecock EM. Neuroglia and pioneer neurons express UNC-6 to provide global and local netrin cues for guiding migrations in C. elegans. Neuron. 1996;16(1):35-46.

3. Lim YS, Wadsworth WG. Identification of domains of netrin UNC-6 that mediate attractive and repulsive guidance and responses from cells and growth cones. J Neurosci. 2002;22(16):7080-7.

4. Hao JC, Yu TW, Fujisawa K, Culotti JG, Gengyo-Ando K, Mitani S, et al. C. elegans Slit Acts in Midline, Dorsal-Ventral, and Anterior-Posterior Guidance via the SAX-3/Robo Receptor. Neuron. 2001;32(1):25-38.

5. Stavoe AK, Nelson JC, Martinez-Velazquez LA, Klein M, Samuel AD, Colon-Ramos DA. Synaptic vesicle clustering requires a distinct MIG-10/Lamellipodin isoform and ABI-1 downstream from Netrin. Genes Dev. 2012;26(19):2206-21.

6. Zallen JA, Yi BA, Bargmann CI. The conserved immunoglobulin superfamily member SAX-3/Robo directs multiple aspects of axon guidance in C. elegans. Cell. 1998;92(2):217-27.

7. Rhiner C, Gysi S, Frohli E, Hengartner MO, Hajnal A. Syndecan regulates cell migration and axon guidance in C. elegans. Development. 2005;132(20):4621-33.
