# Supplementary material for: Functional Requirements for Heparan Sulfate Biosynthesis in Morphogenesis and Nervous System Development in C. elegans
Source: PLoS Genet. 2017 Jan 9;13(1):e1006525. doi: 10.1371/journal.pgen.1006525 (PMC5221758; doi:10.1371/journal.pgen.1006525)
Supplement: S2 Table — (DOCX) [file pgen.1006525.s003.docx]

**S2 Table**. AVM soma and axon guidance defects in *rib-1* and *rib-2* single mutants and in transgenic lines used to rescue with the respective genomic locus.

| **Genotype** | **Transgene** | | | | | **N** | **% Defective** | | | **s.e.p.** |
| --- | --- | --- | --- | --- | --- | --- | --- | --- | --- | --- |
| *zdIs5* |  | | | | | 137 | 1 | | | 0.8 |
| *rib-1(qm32); zdIs5* |  | | | | | 124 | 70 | | | 4.1 |
| *rib-2(qm46); zdIs5* |  | | | | | 159 | 67 | | | 3.7 |
| ***rib-1(+)*-transgenic lines used for rescue of AVM soma and axon guidance** | | | | | | | | | |  |
| *rib-1(qm32); zdIs5; qvEx80* | P*rib-1::rib-1::Venus* | | |  | 84 | | | 0 | 0.0 | |
| *rib-1(qm32); zdIs5; qvEx148* | P*rib-1::rib-1::Venus* | | |  | 123 | | | 3 | 1.5 | |
| ***rib-2(+)­*-transgenic lines used for rescue of AVM soma and axon guidance** | | | | | | | | | | |
| *rib-2(qm46); zdIs5; qmEx329* | | P*rib-2::rib-2(+)* |  | | 141 | | | 5 | 1.8 | |
| *rib-2(qm46); zdIs5; qmEx330* | | P*rib-2::rib-2(+)* |  | | 131 | | | 8 | 2.4 | |

N, number of AVM axons examined. s.e.p., standard error of the proportion.
