# Supplementary material for: Functional Requirements for Heparan Sulfate Biosynthesis in Morphogenesis and Nervous System Development in C. elegans
Source: PLoS Genet. 2017 Jan 9;13(1):e1006525. doi: 10.1371/journal.pgen.1006525 (PMC5221758; doi:10.1371/journal.pgen.1006525)
Supplement: S3 Table — (DOCX) [file pgen.1006525.s004.docx]

**S3 Table**. PVQ guidance defects in *rib-1* and *rib-2* mutants and in transgenic lines to rescue with the respective genomic locus.

| **Genotype** | **Transgene** | **N** | **% Defective** | **s.e.p.** |
| --- | --- | --- | --- | --- |
| *hdIs29* |  | 89 | 11 | 3.3 |
| *rib-1(qm32); hdIs29* |  | 75 | 83 | 4.3 |
| *rib-2(qm46); hdIs29* |  | 78 | 79 | 4.6 |
| ***rib-1(+)-*transgenic lines used for rescue of PVQ guidance** | |  |  |  |
| *rib-1(qm32); hdIs29; qvEx86* | P*rib-1::rib-1* | 33 | 6 | 4.1 |
| *rib-1(qm32); hdIs29; qvEx140* | P*rib-1::rib-1* | 25 | 8 | 5.4 |
| ***rib-2(+)-*transgenic lines used for rescue of PVQ guidance** | |  |  |  |
| *rib-2(qm46); hdIs29; qmEx329* | P*rib-2::rib-2* | 58 | 12 | 4.3 |
| *rib-2(qm46); hdIs29; qmEx330* | P*rib-2::rib-2* | 58 | 16 | 4.8 |

N, number of animals in which PVQL and PVQR axons were examined. s.e.p., standard error of the proportion.
