# Supplementary material for: Functional Requirements for Heparan Sulfate Biosynthesis in Morphogenesis and Nervous System Development in C. elegans
Source: PLoS Genet. 2017 Jan 9;13(1):e1006525. doi: 10.1371/journal.pgen.1006525 (PMC5221758; doi:10.1371/journal.pgen.1006525)
Supplement: S4 Table — (DOCX) [file pgen.1006525.s005.docx]

**S4 Table**. PVQ guidance defects in *rib-1* mutants and in transgenic lines for tissue-specific rescue assays.

| **Genotype** | **Transgene** | | **N** | **% Defective** | **s.e.p.** |
| --- | --- | --- | --- | --- | --- |
| *hdIs29* |  |  | 89# | 11 | 3.3 |
| *rib-1(qm32); hdIs29* |  |  | 75# | 83 | 4.3 |
| *rib-1(qm32); hdIs29; qvEx83* | P*rib-1::rib-1* | | 69 | 16 | 4.4 |
| *rib-1(qm32); hdIs29; qvEx165* | P*rib-1::rib-1* | | 26 | 19 | 7.7 |
| *rib-1(qm32); hdIs29; qvEx153* | P*rgef-1::rib-1* | | 33 | 85 | 6.2 |
| *rib-1(qm32); hdIs29; qvEx154* | P*rgef-1::rib-1* | | 70 | 63 | 5.8 |
| *rib-1(qm32); hdIs29; qvEx155* | P*rgef-1::rib-1* | | 73 | 77 | 4.9 |
| *rib-1(qm32); hdIs29; qvEx43* | P*dpy-7::rib-1* | | 50 | 62 | 6.9 |
| *rib-1(qm32); hdIs29; qvEx157* | P*dpy-7::rib-1* | | 25 | 88 | 6.5 |
| *rib-1(qm32); hdIs29; qvEx158* | P*dpy-7::rib-1* | | 17 | 65 | 11.6 |
| *rib-1(qm32); hdIs29; qvEx47* | P*myo-3::rib-1* | | 58 | 69 | 6.1 |
| *rib-1(qm32); hdIs29; qvEx48* | P*myo-3::rib-1* | | 53 | 58 | 6.8 |
| *rib-1(qm32); hdIs29; qvEx161* | P*myo-3::rib-1* | | 67 | 61 | 6.0 |
| *rib-1(qm32); hdIs29; qvEx162* | P*rgef-1::rib-1,* P*dpy-7::rib-1,* P*myo-3::rib-1* | | 57 | 46 | 6.6 |
| *rib-1(qm32); hdIs29; qvEx163* | P*rgef-1::rib-1,* P*dpy-7::rib-1,* P*myo-3::rib-1* | | 44 | 48 | 7.5 |
| *rib-1(qm32); hdIs29; qvEx164* | P*rgef-1::rib-1,* P*dpy-7::rib-1,* P*myo-3::rib-1* | | 79 | 54 | 5.6 |

N, number of animals in which PVQL and PVQR axons were examined. s.e.p., standard error of the proportion.

#, Data from **S3 Table**.
