# Supplementary material for: Functional Requirements for Heparan Sulfate Biosynthesis in Morphogenesis and Nervous System Development in C. elegans
Source: PLoS Genet. 2017 Jan 9;13(1):e1006525. doi: 10.1371/journal.pgen.1006525 (PMC5221758; doi:10.1371/journal.pgen.1006525)
Supplement: S5 Table — (DOCX) [file pgen.1006525.s006.docx]

**S5 Table**. AVM axon guidance of *rib-1* mutants and in transgenic lines used for tissue-specific rescue assays.

| **Genotype** | **Transgene** | | **N** | **% Defective** | **s.e.p.** |
| --- | --- | --- | --- | --- | --- |
| *zdIs5* | |  | 136 | 1 | 0.9 |
| *rib-1(qm32); zdIs5* | |  | 345 | 53 | 2.7 |
| *rib-1(qm32); zdIs5; qvEx131* | P*rib-1::rib-1* | | 28 | 4 | 3.7 |
| *rib-1(qm32); zdIs5; qvEx81* | P*rib-1::rib-1* | | 61 | 5 | 2.8 |
| *rib-1(qm32); zdIs5; qvEx132* | P*rib-1::rib-1* | | 33 | 0 | 0.0 |
| *rib-1(qm32); zdIs5; qvEx133* | P*mec-7::rib-1* | | 139 | 14 | 2.9 |
| *rib-1(qm32); zdIs5; qvEx85* | P*mec-7::rib-1* | | 72 | 13 | 4.0 |
| *rib-1(qm32); zdIs5; qvEx101* | P*mec-7::rib-1* | | 154 | 12 | 2.6 |
| *rib-1(qm32); zdIs5; qvEx102* | P*mec-7::rib-1* | | 242 | 10 | 1.9 |
| *rib-1(qm32); zdIs5; qvEx95* | P*dpy-7::rib-1* | | 70 | 27 | 5.3 |
| *rib-1(qm32); zdIs5; qvEx136* | P*dpy-7::rib-1* | | 97 | 31 | 4.7 |
| *rib-1(qm32); zdIs5; qvEx137* | P*dpy-7::rib-1* | | 73 | 27 | 5.2 |
| *rib-1(qm32); zdIs5; qvEx139* | P*dpy-7::rib-1* | | 58 | 28 | 5.9 |
| *rib-1(qm32); zdIs5; qvEx134* | P*myo-3::rib-1* | | 52 | 27 | 6.2 |
| *rib-1(qm32); zdIs5; qvEx141* | P*myo-3::rib-1* | | 60 | 33 | 6.1 |
| *rib-1(qm32); zdIs5; qvEx135* | P*myo-3::rib-1* | | 57 | 35 | 6.3 |
| *rib-1(qm32); zdIs5; qvEx94* | P*myo-3::rib-1* | | 70 | 39 | 5.8 |

N, number of AVM axons examined. s.e.p., standard error of the proportion.
