# Supplementary material for: Functional Requirements for Heparan Sulfate Biosynthesis in Morphogenesis and Nervous System Development in C. elegans
Source: PLoS Genet. 2017 Jan 9;13(1):e1006525. doi: 10.1371/journal.pgen.1006525 (PMC5221758; doi:10.1371/journal.pgen.1006525)
Supplement: S6 Table — (DOCX) [file pgen.1006525.s007.docx]

**S6 Table**. AVM axon guidance defects in *rib-1* and *rib-2* mutants in combination with AVM guidance pathway mutants.

| **Genotype** | **N** | **% AVM defective** | **s.e.p.** |
| --- | --- | --- | --- |
| *zdIs5* | 170 | 0.6 | 0.6 |
| *rib-1(qm32);zdIs5* | 345# | 53 | 2.7 |
| *rib-2(qm46); zdIs5* | 339 | 51 | 2.7 |
| *unc-6(e78); zdIs5* | 191 | 24 | 3.1 |
| *rib-1(qm32); unc-6(e78); zdIs5* | 79 | 81 | 4.4 |
| *rib-2(qm46); unc-6(e78); zdIs5* | 189 | 80 | 2.9 |
| *slt-1(eh15); zdIs5* | 195 | 47 | 3.6 |
| *rib-1(qm32); slt-1(eh15); zdIs5* | 201 | 74 | 3.1 |
| *rib-2(qm46); slt-1(eh15); zdIs5* | 180 | 82 | 2.9 |
| P*myo-3::slt-1* | 157 | 17 | 3.0 |
| *rib-1(qm32);* P*myo-3::slt-1* | 199 | 73 | 3.1 |
| *rib-2(qm46);* P*myo-3::slt-1* | 198 | 77 | 3.0 |

N, number of AVM axons examined. s.e.p., standard error of the proportion.

#, Data from **S4 Table**.
