# Supplementary material for: Functional Requirements for Heparan Sulfate Biosynthesis in Morphogenesis and Nervous System Development in C. elegans
Source: PLoS Genet. 2017 Jan 9;13(1):e1006525. doi: 10.1371/journal.pgen.1006525 (PMC5221758; doi:10.1371/journal.pgen.1006525)
Supplement: S7 Table — (DOCX) [file pgen.1006525.s008.docx]

**S7 Table.** PVM dorsal guidance defects quantified in wild-type and mutant strains with or without misexpression of *unc-5* in the PVM neuron using transgene *evIs25* P*mec-7::unc-5*.

| **Genotype** | **N** | **% Dorsal PVM axon ^** | **s.e.p.** |
| --- | --- | --- | --- |
| **PVM controls** | | | |
| *zdIs5* | 52^#^ | **0** | 0.0 |
| *rib-1(qm32); zdIs5* | 110 | **4** | 1.9 |
| *rib-2(qm46); zdIs5* | 82 | **0** | 0.0 |
| *unc-6(ev400); zdIs5* | 44^#^ | **0** | 0.0 |
| *unc-40(e271) zdIs5* | 30^#^ | **0** | 0.0 |
| *slt-1(eh15); zdIs5* | 60^#^ | **0** | 0.0 |
| *sax-3(ky123); zdIs5* | 41^#^ | **0** | 0.0 |
| *sdn-1(zh20); zdIs5* | 58^#^ | **0** | 0.0 |
| **Strains with *evIs25* P*mec-7::unc-5*** | | | |
| *evIs25; zdIs5* | 228^#^ | **66** | 3.1 |
| *rib-1(qm32); evIs25; zdIs5* | 230 | **51** | 3.3 |
| *rib-2(qm46); evIs25; zdIs5* | 254 | **43** | 3.1 |
| *unc-6(ev400) evIs25; zdIs5* | 41^#^ | **0** | 0.0 |
| *unc-40(e271)* *zdIs5; evIs25* | 191^#^ | **18** | 2.8 |
| *slt-1(eh15)* *evIs25*; *zdIs5* | 212^#^ | **63** | 3.3 |
| *sax-3(ky123)* *evIs25*; *zdIs5* | 201^#^ | **66** | 3.3 |
| *sdn-1(zh20) evIs25; zdIs5* | 259^#^ | **64** | 3.0 |

N, number of AVM axons examined. s.e.p., standard error of the proportion.

^ PVM axons normally never extend dorsally, not even in the complete absence of the *slt-1*/slit and the *unc-6*/netrin guidance pathways in *unc-6 slt-1* double null mutants, where axons defective in guidance extend *anteriorly*. Dorsal axon extension is only observed with *unc-5*/UNC5 ectopic expression (and in 4% of *rib-1(qm32)* mutants), which overpowers the endogenous signaling mechanism within PVM and thus forces its axon to extend dorsally. This highlights the power of this ectopic-*unc-5*-expression system to uncover molecules specifically involved in *unc-6/*netrin signaling through the *unc-5/*UNC5 receptor, independently of other endogenous signals.

^#^ Data from article by Blanchette CR, Perrat PN, Thackeray A, Benard CY. Glypican Is a Modulator of Netrin-Mediated Axon Guidance. PLoS Biol. 2015;13(7):e1002183.
