# Supplementary material for: Functional Requirements for Heparan Sulfate Biosynthesis in Morphogenesis and Nervous System Development in C. elegans
Source: PLoS Genet. 2017 Jan 9;13(1):e1006525. doi: 10.1371/journal.pgen.1006525 (PMC5221758; doi:10.1371/journal.pgen.1006525)
Supplement: S8 Table — (DOCX) [file pgen.1006525.s009.docx]

**S8 Table**. AVM axon guidance defects in HS-modifying enzyme mutants in combination with AVM guidance pathway mutants.

| **Genotype** | **N** | **% AVM defective** | **s.e.p.** |
| --- | --- | --- | --- |
| *zdIs5* | 114 | 1 | 0.9 |
| *hse-5(tm472); zdIs5* | 318 | 8 | 1.5 |
| *hst-2(ok595); zdIs5* | 199 | 6 | 1.7 |
| *hst-6(ok273); zdIs5* | 196 | 2 | 1.0 |
|  |  |  |  |
| *hse-5(tm472); hst-2(ok595); zdIs5* | 240 | 13 | 2.2 |
| *hse-5(tm472); hst-6(ok273); zdIs5* | 182 | 41 | 3.6 |
| *hst-2(ok595) hst-6(ok273); zdIs5* | 211 | 47 | 3.4 |
| *hse-5(tm472); hst-2(ok595) hst-6(ok273); zdIs5* | 239 | 46 | 3.2 |
|  |  |  |  |
| *unc-6(ev400); zdIs5* | 190 | 44 | 3.6 |
| *hse-5(tm472); unc-6(ev400); zdIs5* | 262 | 72 | 2.8 |
| *hst-2(ok595) unc-6(ev400); zdIs5* | 200 | 74 | 3.1 |
| *hst-6(ok273) unc-6(ev400); zdIs5* | 223 | 67 | 3.1 |
|  |  |  |  |
| *slt-1(eh15); zdIs5* | 106 | 49 | 4.9 |
| *hse-5(tm472); slt-1(eh15); zdIs5* | 195 | 79 | 2.9 |
| *hst-2(ok595) slt-1(eh15); zdIs5* | 207 | 85 | 2.5 |
| *hst-6(ok273) slt-1(eh15); zdIs5* | 193 | 73 | 3.2 |
| *hse-5(tm472); hst-2(ok595) hst-6(ok273) slt-1(eh15)* | 78 | 91 | 3.2 |

N, number of AVM axons examined. s.e.p., standard error of the proportion.
