# Supplementary material for: Functional Requirements for Heparan Sulfate Biosynthesis in Morphogenesis and Nervous System Development in C. elegans
Source: PLoS Genet. 2017 Jan 9;13(1):e1006525. doi: 10.1371/journal.pgen.1006525 (PMC5221758; doi:10.1371/journal.pgen.1006525)
Supplement: S9 Table — (DOCX) [file pgen.1006525.s010.docx]

**S9 Table**. List of strains used.

| **Strain** | **Genotype** | | **Transgene** | **Reference** | |
| --- | --- | --- | --- | --- | --- |
| N2 |  | |  | [1] | |
| LE311 | *lqIs4 III* | | P*ceh-10::gfp* | [2] | |
|  | *hdIs29 V* | | P*odr-2::cfp;* P*sra-6::DsRed2* | [3] | |
| MT4005 | *zdIs5 I* | | P*mec-4::gfp; lin-15(+)* | [4] | |
| SK4013 | *zdIs13 IV* | | P*tph-1::gfp* | [4] | |
| VQ84 | *vsIs48 X; ufIs34* | | P*unc-17::gfp;* P*unc-47::mCherry* | [5, 6] | |
| NW1229 | *evIs111 III* | | P*rgef-1::gfp; dpy-20(+)* | [7] | |
| OH1360 | *bgIs312* | | P*pes-6::gfp* | [8] | |
| ***rib-1* and *rib-2* alone and with AVM guidance factors** | | | | | |
| VQ344 | *rib-1(qm32) IV; lqIs4 III* | |  | This study | |
| VQ355 | *rib-2(qm46) III; lqIs4 III* | |  | This study | |
| VQ196 | *rib-1(qm32) IV; hdIs29 V* | |  | This study | |
| VQ212 | *rib-2(qm46) III; hdIs29 V* | |  | This study | |
| VQ252 | *rib-1(qm32) IV; zdIs5 I* | |  | This study | |
| VQ200 | *rib-2(qm46) III; zdIs5 I* | |  | This study | |
| VQ661 | *rib-1(qm32 ) zdIs13 IV* | |  | This study | |
| VQ402 | *rib-2(qm46) III; zdIs13 IV* | |  | This study | |
| VQ202 | *rib-1(qm32) IV; vsIs48; ufIs34* | |  | This study | |
| VQ208 | *rib-2(qm46) III; vsIs48; ufIs34* | |  | This study | |
| VQ209 | *rib-1(qm32) IV; evIs111 III* | |  | This study | |
| VQ213 | *rib-2(qm46) evIs111 III* | |  | This study | |
| VQ342 | *rib-1(qm32) IV; bgIs312* | |  | This study | |
| VQ343 | *rib-2(qm46) III; bgIs312* | |  | This study | |
| VQ230 | *rib-1(qm32)/*  *unc-24(e138) dpy-20(e1282ts) IV;*  *hdIs29 V* | |  | This study | |
| VQ265 | *rib-1(qm32)/*  *unc-24(e138) dyp-20(e1282ts) IV;*  *zdIs5 I* | |  | This study | |
| VQ465 | *rib-1(qm32) IV; unc-6(e78) X; zdIs5 I* | |  | This study | |
| VQ449 | *rib-2(qm46) IV; unc-6(e78) X; zdIs5 I* | |  | This study | |
| VQ405 | *rib-1(qm32) IV; slt-1(eh15) X; zdIs5 I* | |  | This study | |
| VQ404 | *rib-2(qm46) IV; slt-1(eh15) X; zdIs5 I* | |  | This study | |
| VQ443 | *rib-1(qm32) IV; kyIs209 X; zdIs5 I* | |  | This study | |
| VQ456 | *rib-2(qm46) IV; kyIs209 X; zdIs5 I* | |  | This study | |
| **HS-modifying enzymes and AVM axon guidance factors** | | | | | |
| VQ551 | *hse-5(tm472) III; zdIs5 I* | |  | This study | |
| VQ547 | *hst-2(ok595) X; zdIs5 I* | |  | This study | |
| VQ546 | *hst-6(ok273) X; zdIs5 I* | |  | This study | |
| VQ569 | *hse-5(tm472) III; hst-2(ok595) X; zdIs5 I* | |  | This study | |
| VQ560 | *hse-5(tm472) III; hst-6(ok273) X; zdIs5 I* | |  | This study | |
| VQ571 | *hst-2(ok595) hst-6(ok273) X; zdIs5 I* | |  | This study | |
| VQ599 | *hst-2(ok595) hst-6(ok273) X;*  *hse-5(tm472) III; zdIs5 I* | |  | This study | |
| VQ395 | *unc-6(e78) X; zdIs5 I* | |  | [9] | |
| VQ577 | *hse-5(tm472) III; unc-6(ev400) X; zdIs5 I* | |  | This study | |
| VQ573 | *hst-2(ok595) unc-6(ev400) X; zdIs5 I* | |  | This study | |
| VQ579 | *hst-6(ok273) unc-6(ev400) X; zdIs5 I* | |  | This study | |
| VQ401 | *slt-1(eh15) X; zdIs5 I* | |  | [9] | |
| VQ427 | *kyIs209 X; zdIs5 I* | |  | [9] | |
| VQ602 | *hse-5(tm472) III; slt-1(eh15) X; zdIs5 I* | |  | This study | |
| VQ604 | *hst-2(ok595) slt-1(eh15) X; zdIs5 I* | |  | This study | |
| VQ606 | *hst-6(ok273) slt-1(eh15) X; zdIs5 I* | |  | This study | |
| VQ616 | *hse-5(tm472) III; hst-2(ok595)*  *hst-6(ok273) slt-1(eh15)X; zdIs5 I* | |  | This study | |
| **LON-2::GFP and SDN-1::GFP strains** | | | | | |
| WS3404 | *opIs171* | | P*sdn-1::sdn-1::gfp; lin-15(+)* | [10] | |
| VQ450 | *rib-1(qm32) IV; opIs171* | | P*sdn-1::sdn-1::gfp; lin-15(+)* | This study | |
| TLG257 | *lon-2(e678) X; texEx164* | | Plasmid HW483 (P*lon-2::lon-2::gfp),*  P*ttx-3::mCherry* | [11] | |
| TLG199 | *lon-2(e678) X; texEx144* | | pSBL3SG006 (P*lon-2::*LON-2ΔGAG  ::*gfp*), P*ttx-3::mCherry* | [11] | |
| VQ525 | *rib-1(qm32) IV; texEx164* | | Plasmid HW483 (P*lon-2::lon-2::gfp),*  P*ttx-3::mCherry* | This study | |
| VQ528 | *rib-2(qm46) III; texEx164* | | Plasmid HW483 (P*lon-2::lon-2::gfp),*  P*ttx-3::mCherry* | This study | |
| **Transgenic Lines** | |  |  | |  |
| VQ370 | *rib-1(qm32) IV; zdIs5 I; qvEx80* | | pCB221 (P*rib-1::rib-1::Venus*),  P*ttx-3::mCherry*, pRF4. Line #1 | This study | |
| VQ684 | *rib-1(qm32) IV; zdIs5 I; qvEx148* | | pCB221 (P*rib-1::rib-1::Venus*),  P*ttx-3::mCherry*, pRF4. Line #2 | This study | |
| VQ502 | *rib-2(qm46) III; zdIs5 I; qmEx329* | | PCR product of bases 604..6196 of  cosmid K01G5, pRF4. Line #1 | This study | |
| VQ503 | *rib-2(qm46) III; zdIs5 I; qmEx330* | | PCR product of bases 604..6196 of  cosmid K01G5, pRF4. Line #2 | This study | |
| VQ381 | *rib-1(qm32) IV; hdIs29 V; qvEx86* | | pCB221 (P*rib-1::rib-1 ::Venus*),  P*ttx-3::mCherry*, pRF4. Line #1 | This study | |
| VQ673 | *rib-1(qm32) IV; hdIs29 V; qvEx140* | | pCB221 (P*rib-1::rib-1::Venus*),  P*ttx-3::mCherry*, pRF4. Line #2 | This study | |
| VQ504 | *rib-2(qm46) III; hdIs29 V; qmEx329* | | PCR product of bases 604…6196 of  cosmid K01G5, pRF4. Line #1 | This study | |
| VQ505 | *rib-2(qm46) III; hdIs29 V; qmEx330* | | PCR product of bases 604…6196 of  cosmid K01G5, pRF4. Line #2 | This study | |
| VQ663 | *rib-1(qm32) IV; zdIs5 I; qvEx131* | | pCB225 (P*rib-1::rib-1*), P*ttx-3::mCherry,*  pRF4. Line #1 | This study | |
| VQ376 | *rib-1(qm32) IV; zdIs5 I; qvEx81* | | pCB225 (P*rib-1::rib-1*), P*ttx-3::mCherry,*  pRF4. Line #2 | This study | |
| VQ664 | *rib-1(qm32) IV; zdIs5 I; qvEx132* | | pCB225 (P*rib-1::rib-1*), P*ttx-3::mCherry,*  pRF4. Line #3 | This study | |
| VQ665 | *rib-1(qm32) IV; zdIs5 I; qvEx133* | | pCB204 (P*mec-7::rib-1*), P*ceh-22::gfp,*  pRF4. Line #1 | This study | |
| VQ334 | *rib-1(qm32) IV; zdIs5 I;*  *qvEx85* | | pCB204 (P*mec-7::rib-1*), P*ceh-22::gfp,*  pRF4. Line #2 | This study | |
| VQ508 | *rib-1(qm32) IV; zdIs5 I; qvEx101* | | pCB204 (P*mec-7::rib-1*), pRF4, P*rgef-1*  ::*DsRed2*. Line #3 | This study | |
| VQ509 | *rib-1(qm32) IV; zdIs5 I; qvEx102* | | pCB204 (P*mec-7::rib-1*), pRF4, P*rgef-1*  ::*DsRed2*. Line #4 | This study | |
| VQ667 | *rib-1(qm32) IV; zdIs5 I; qvEx134* | | pCB196 (P*myo-3::rib-1*), P*ttx-3*  *::mCherry*, pRF4. Line #1 | This study | |
| VQ666 | *rib-1(qm32) IV; zdIs5 I; qvEx141* | | pCB196 (P*myo-3::rib-1*), P*ttx-3*  *::mCherry*, pRF4. Line #2 | This study | |
| VQ668 | *rib-1(qm32) IV; zdIs5 I; qvEx135* | | pCB196 (P*myo-3::rib-1*), P*ttx-3*  *::mCherry*, pRF4. Line #3 | This study | |
| VQ487 | *rib-1(qm32) IV; zdIs5 I;*  *qvEx94* | | pCB196 (P*myo-3::rib-1*), P*ttx-3*  *::mCherry*, pRF4. Line #4 | This study | |
| VQ488 | *rib-1(qm32) IV; zdIs5 I;*  *qvEx95* | | pCB186 (P*dpy-7::rib-1*), P*ttx-3*  *::mCherry,* pRF4. Line #1 | This study | |
| VQ669 | *rib-1(qm32) IV; zdIs5 I; qvEx136* | | pCB186 (P*dpy-7::rib-1*), P*ttx-3*  *::mCherry,* pRF4. Line #2 | This study | |
| VQ670 | *rib-1(qm32) IV; zdIs5 I; qvEx137* | | pCB186 (P*dpy-7::rib-1*), P*ttx-3*  *::mCherry,* pRF4. Line #3 | This study | |
| VQ672 | *rib-1(qm32) IV; zdIs5 I; qvEx139* | | pCB186 (P*dpy-7::rib-1*), P*ttx-3*  *::mCherry,* pRF4. Line #4 | This study | |
| VQ391 | +; *qvEx90* | | pCB78 (P*rib-1::gfp*), pRF4. Line #1 | This study | |
| VQ204 | +; *qvEx36* | | pCB78 (P*rib-1::gfp*), P*ttx-3::mCherry*,  pBSK+. Line #2 | This study | |
| VQ205 | +; *qvEx37* | | pCB78 (P*rib-1::gfp*), P*ttx-3::mCherry*,  pBSK+. Line #3 | This study | |
| VQ356 | +; *qvEx79* | | pCB221 (P*rib-1::rib-1 ::Venus*), pRF4,  P*unc-122::rfp.* Line #1 | This study | |
| VQ379 | +; *qvEx84* | | pCB221 (P*rib-1::rib-1 ::Venus*), pRF4.  Line #2 | This study | |
| VQ694 | *rib-1(qm32) IV; hdIs29 V; qvEx153* | | pCB199 (P*rgef-1::rib-1*), P*ttx-3*  *::mCherry*, pRF4. Line #1 | This study | |
| VQ695 | *rib-1(qm32) IV; hdIs29 V; qvEx154* | | pCB199 (P*rgef-1::rib-1*), P*ttx-3*  *::mCherry*, pRF4. Line #2 | This study | |
| VQ696 | *rib-1(qm32) IV; hdIs29 V; qvEx155* | | pCB199 (P*rgef-1::rib-1*), P*ttx-3*  *::mCherry*, pRF4. Line #3 | This study | |
| VQ253 | *rib-1(qm32) IV; hdIs29 V; qvEx43* | | pCB186 (P*dpy-7::rib-1*), P*ttx-3*  *::mCherry*, pRF4. Line #1 | This study | |
| VQ698 | *rib-1(qm32) IV; hdIs29 V; qvEx157* | | pCB186 (P*dpy-7::rib-1*), P*ttx-3*  *::mCherry*, pRF4. Line #2 | This study | |
| VQ699 | *rib-1(qm32) IV; hdIs29 V; qvEx158* | | pCB186 (P*dpy-7::rib-1*), P*ttx-3*  *::mCherry*, pRF4. Line #3 | This study | |
| VQ260 | *rib-1(qm32) IV; hdIs29 V; qvEx47* | | pCB196 (P*myo-3::rib-1*), P*ttx-3*  *::mCherry*, pRF4. Line #1 | This study | |
| VQ261 | *rib-1(qm32) IV; hdIs29 V; qvEx48* | | pCB196 (P*myo-3::rib-1*), P*ttx-3*  *::mCherry*, pRF4. Line #2 | This study | |
| VQ702 | *rib-1(qm32) IV; hdIs29 V; qvEx161* | | pCB196 (P*myo-3::rib-1*), P*ttx-3*  *::mCherry*, pRF4. Line #3 | This study | |
| VQ703 | *rib-1(qm32) IV; hdIs29 V; qvEx162* | | pCB199 (P*rgef-1::rib-1*)*,* pCB186  (P*dpy-7::rib-1*), pCB196 (P*myo-3::rib-1*),  P*ttx-3::mCherry*, pRF4. Line #1 | This study | |
| VQ704 | *rib-1(qm32) IV; hdIs29 V; qvEx163* | | pCB199 (P*rgef-1::rib-1*)*,* pCB186  (P*dpy-7::rib-1*), pCB196 (P*myo-3::rib-1*),  P*ttx-3::mCherry*, pRF4. Line #2 | This study | |
| VQ705 | *rib-1(qm32) IV; hdIs29 V; qvEx164* | | pCB199 (P*rgef-1::rib-1*)*,* pCB186  (P*dpy-7::rib-1*), pCB196 (P*myo-3::rib-1*),  P*ttx-3::mCherry*, pRF4. Line #3 | This study | |
| ***evIs25* strains and controls** | | | | | |
| VQ396 | *unc-6(ev400) X; zdIs5 I* | |  | [9] | |
| VQ470 | *unc-40(e271) zdIs5 I* | |  | [9] | |
| VQ473 | *sax-3(k123) X; zdIs5 I* | |  | [9] | |
| VQ423 | *sdn-1(zh20) X; zdIs5 I* | |  | [9] | |
| VQ536 | *evIs25 X; zdIs5 I* | |  | [9] | |
| VQ538 | *unc-6(ev400) evIs25 X; zdIs5 I* | |  | [9] | |
| VQ540 | *unc-40(e271)* *zdIs5 I; evIs25 X* | |  | [9] | |
| VQ556 | *slt-1(eh15)* *evIs25 X*; *zdIs5 I* | |  | [9] | |
| VQ557 | *sax-3(ky123)* *evIs25 X*; *zdIs5 I* | |  | [9] | |
| VQ572 | *sdn-1(zh20) evIs25 X; zdIs5 I* | |  | [9] | |
| VQ554 | *rib-1(qm32) IV; evIs25 X; zdIs5 I* | |  | This study | |
| VQ539 | *rib-2(qm46) III; evIs25 X; zdIs5 I* | |  | This study | |

1. Brenner S. The genetics of Caenorhabditis elegans. Genetics. 1974;77(1):71-94.

2. Tsalik EL, Niacaris T, Wenick AS, Pau K, Avery L, Hobert O. LIM homeobox gene-dependent expression of biogenic amine receptors in restricted regions of the C. elegans nervous system. Dev Biol. 2003;263(1):81-102. PubMed PMID: 14568548.

3. Schmitz C, Wacker I, Hutter H. The Fat-like cadherin CDH-4 controls axon fasciculation, cell migration and hypodermis and pharynx development in Caenorhabditis elegans. Dev Biol. 2008;316(2):249-59. doi: 10.1016/j.ydbio.2008.01.024. PubMed PMID: 18328472.

4. Clark SG, Chiu C. C. elegans ZAG-1, a Zn-finger-homeodomain protein, regulates axonal development and neuronal differentiation. Development. 2003;130(16):3781-94. PubMed PMID: 12835394.

5. Chase DL, Pepper JS, Koelle MR. Mechanism of extrasynaptic dopamine signaling in Caenorhabditis elegans. Nat Neurosci. 2004;7(10):1096-103. PubMed PMID: 15378064.

6. Petrash HA, Philbrook A, Haburcak M, Barbagallo B, Francis MM. ACR-12 ionotropic acetylcholine receptor complexes regulate inhibitory motor neuron activity in Caenorhabditis elegans. J Neurosci. 2013;33(13):5524-32. doi: 10.1523/JNEUROSCI.4384-12.2013. PubMed PMID: 23536067; PubMed Central PMCID: PMC3645261.

7. Altun-Gultekin Z, Andachi Y, Tsalik EL, Pilgrim D, Kohara Y, Hobert O. A regulatory cascade of three homeobox genes, ceh-10, ttx-3 and ceh-23, controls cell fate specification of a defined interneuron class in C. elegans. Development. 2001;128(11):1951-69. PubMed PMID: 11493519.

8. Berry KL, Bulow HE, Hall DH, Hobert O. A C. elegans CLIC-like protein required for intracellular tube formation and maintenance. Science. 2003;302(5653):2134-7. PubMed PMID: 14684823.

9. Blanchette CR, Perrat PN, Thackeray A, Benard CY. Glypican Is a Modulator of Netrin-Mediated Axon Guidance. PLoS Biol. 2015;13(7):e1002183. doi: 10.1371/journal.pbio.1002183. PubMed PMID: 26148345; PubMed Central PMCID: PMCPMC4493048.

10. Rhiner C, Gysi S, Frohli E, Hengartner MO, Hajnal A. Syndecan regulates cell migration and axon guidance in C. elegans. Development. 2005;132(20):4621-33. doi: 10.1242/dev.02042. PubMed PMID: 16176946.

11. Taneja-Bageshwar S, Gumienny TL. Two functional domains in C. elegans glypican LON-2 can independently inhibit BMP-like signaling. Dev Biol. 2012;371(1):66-76. doi: 10.1016/j.ydbio.2012.08.006. PubMed PMID: 22922164.
