# Supplementary material for: Functional Requirements for Heparan Sulfate Biosynthesis in Morphogenesis and Nervous System Development in C. elegans
Source: PLoS Genet. 2017 Jan 9;13(1):e1006525. doi: 10.1371/journal.pgen.1006525 (PMC5221758; doi:10.1371/journal.pgen.1006525)
Supplement: S10 Table — (DOCX) [file pgen.1006525.s011.docx]

**S10 Table.** List of primers used.

| **Gene** | **Primer** | **Sequence** | **PCR product**  **(bp)** | **Cosmid Coordinates** |
| --- | --- | --- | --- | --- |
| ***rib-1(qm32)* sequencing** | |  |  |  |
|  | oCB1026 | gggtgcgtaaggagatgagg | 456 | F12F6 39341…39360, forward |
|  | oCB1027 | ggcaaccagccatcacagcc |  | F12F6 39797…39816, reverse |
| ***rib-2(qm46)* sequencing** | |  |  |  |
|  | oCB1028 | caacttatcggatcttcaacc | 437 | K01G5 4053…4073, forward |
|  | oCB1029 | ttccagcggtccaaggagg |  | K01G5 4508...4490, reverse |
| ***slt-1(eh15)*** |  |  |  |  |
| Mutant specific | oCB919 | tatgacgtgttccggaaacc | 467 | C26G2 26145…26164, forward |
|  | oCB920 | atttctctaatacgggtagc |  | C26G2 28538…28557, reverse |
| Wild-type specific | oCB922 | tctcaattctaacatccatgtc | 339 | C26G2 28199…28220, forward |
|  | oCB920 | atttctctaatacgggtagc |  | C26G2 28538…28557, reverse |
| ***sax-3(ky123)*** |  |  |  |  |
| Mutant specific | oCB1038 | agaatgtggctctctagtcc | ~330 | ZK377 15840…15859, forward |
|  | oCB1039 | tcgtttccgcgcattcagtc |  | ZK377 16580…16599, reverse |
| Wild-type specific | oCB1038 | agaatgtggctctctagtcc | 527 | ZK377 15840…15859, forward |
|  | oCB1042 | agcttcggattactgcttgc |  | ZK377 16348…16367, reverse |
| ***sdn-1(zh20)*** |  |  |  |  |
| Mutant specific | oCB837 | aaagagatgccggtcaggtg | 410 | F57C7 28510…28529, forward |
|  | oCB842 | aatggacgggatgagtgtcc |  | F57C7 26861…26880, reverse |
| Wild-type specific | oCB837 | aaagagatgccggtcaggtg | 293 | F57C7 28510…28529, forward |
|  | oCB876 | cttcagattcgagcctgctttgc |  | F57C7 28237…28259, reverse |
| ***evIs25:* Detection of insertion P*mec-7::unc-5*** | | | | |
|  | oCB933 | ttgtcagtcgagcctcaagg | ~631 |  |
|  | oCB966 | tccactgtctgataatctgg |  |  |
| ***kyIs209:* Detection of insertion P*myo-3::slt-1*** | | | | |
|  | oCB945 | tcattcgggatattttgtgg | 592 |  |
|  | oCB950 | aagaagaagcatgcttctgg |  |  |
| ***hse-5(tm472)*** |  |  |  |  |
| Mutant specific | oCB1055 | atcgtgtacgatgtgtcagc | 546 | B0285 15457…15476, forward |
|  | oCB1056 | attcgcctcatacggtttcc |  | B0285 16217…16236, reverse |
| Wild-type specific | oCB1055 | atcgtgtacgatgtgtcagc | 779 | B0285 15457…15476, forward |
|  | oCB1057 | aactttctctcggcaattg |  | B0285 17233…17251, reverse |
| ***hst-2(ok595)*** |  |  |  |  |
| Mutant specific | oCB1052 | tattacaacatggacggagc | 692 | C34F6 17093…17112, forward |
|  | oCB1054 | aacattatgcgcatgaacgc |  | C34F6 15085…15104, reverse |
|  |  |  |  |  |
| Wild-type specific | oCB1052 | tattacaacatggacggagc | 486 | C34F6 17093…17112, forward |
|  | oCB1053 | ttagcagtgattcaattacg |  | C34F6 16626…16645, reverse |
| ***hst-6(ok273)*** |  |  |  |  |
| Mutant specific | oCB1049 | ttagacgtggctgttctcac | 723 | Y34B4A 39244…39263, forward |
|  | oCB1051 | tgtgagtctgttaagggtgg |  | Y34B4A 41012…41031, reverse |
| Wild-type specific | oCB1049 | ttagacgtggctgttctcac | 804 | Y34B4A 39244…39263, forward |
|  | oCB1212 | agaaatgttgtgtagaagtag |  | Y34B4A 40028…40048, reverse |
