# Supplementary figures and images for: Functional Requirements for Heparan Sulfate Biosynthesis in Morphogenesis and Nervous System Development in C. elegans
Source: PLoS Genet. 2017 Jan 9;13(1):e1006525. doi: 10.1371/journal.pgen.1006525 (PMC5221758; doi:10.1371/journal.pgen.1006525)

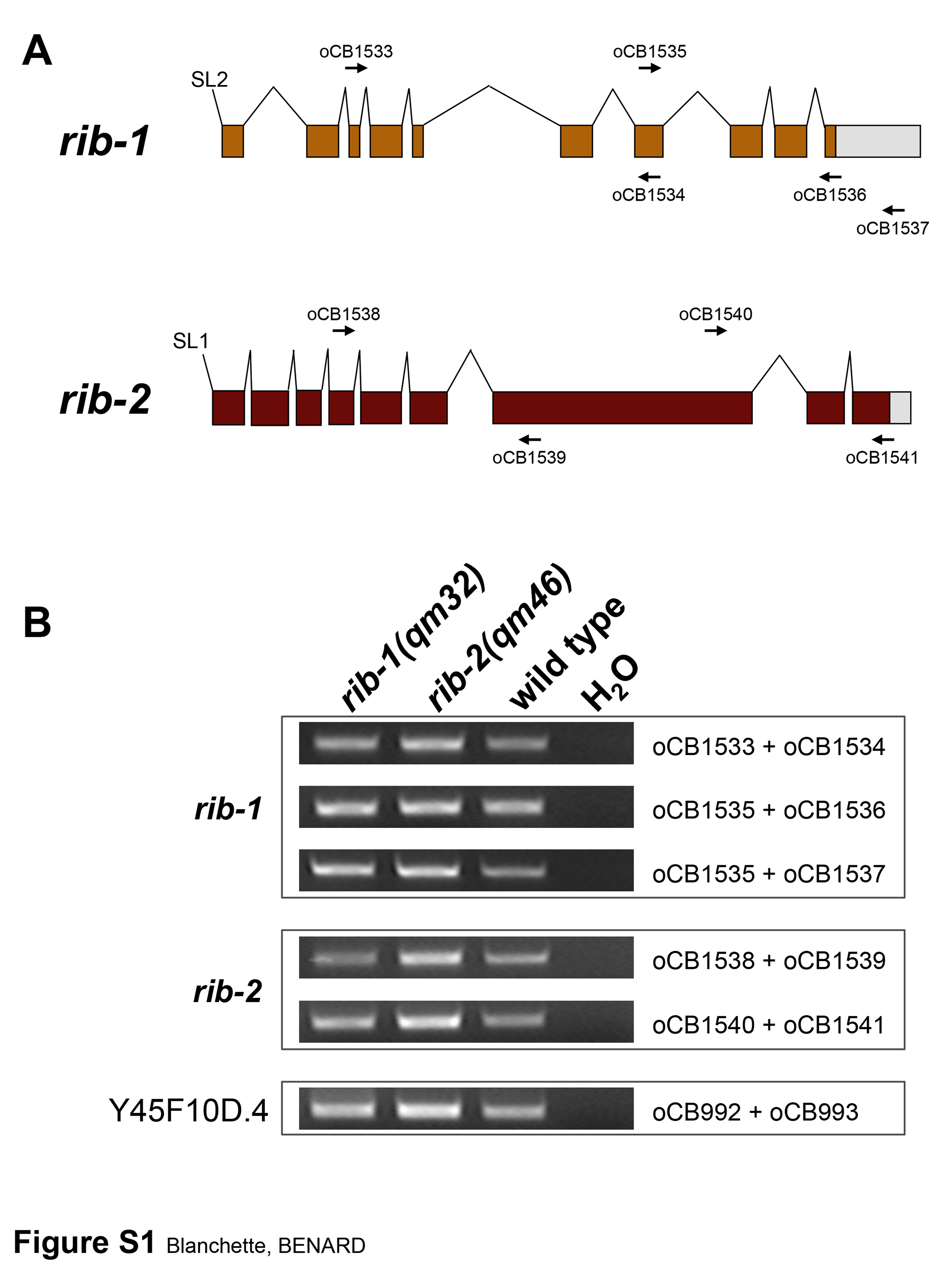

Supplement: S1 Fig — A. Schematic of the gene structure of rib-1 and rib-2 (boxes are exons and lines are introns) and the primers used for RT-PCR analysis. B. RT-PCR performed on wild type, rib-1(qm32)m-/-z-/- and rib-2(qm46)m-/-z-/- mutants. rib-1 and rib-2 transcript levels are comparable in the wild type and rib-1(qm32)m-/-z-/- and rib-2(qm46)m-/-z-/- mutants. (TIF) [file pgen.1006525.s012.tif]

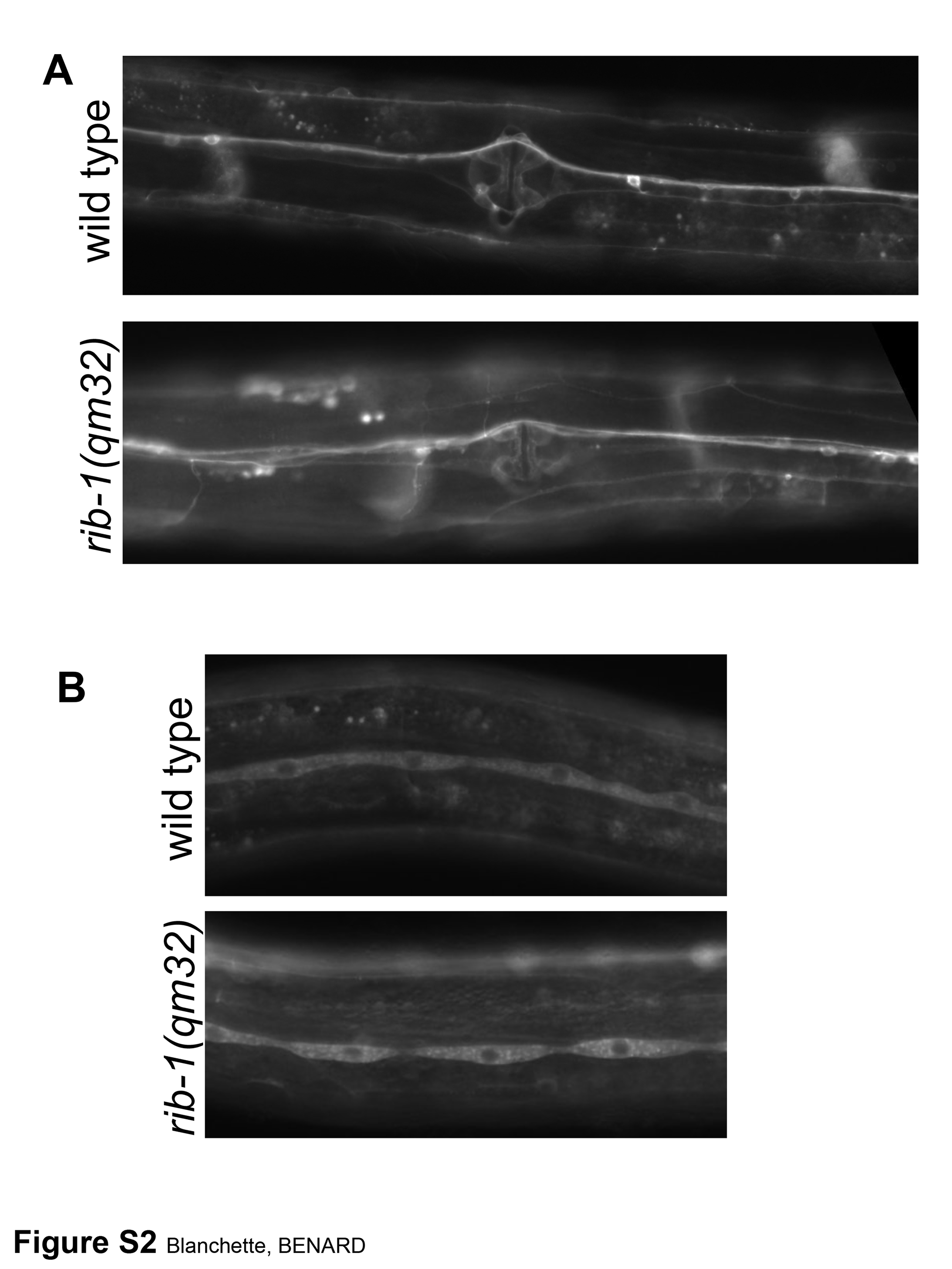

Supplement: S2 Fig — A. Ventral view of mid body region of fourth larval stage animals; the asterisk denotes the vulva, and the arrows indicate the ventral nerve cord. B. Lateral view of fourth larval stage animals showing lateral hypodermal cells (seam cells). Analysis of SDN-1::GFP in rib-2(qm46)m-/- z-/- mutants was not possible as a strain of rib-2(qm46) carrying opIs171 [SDN-1::GFP] could not be built. (TIF) [file pgen.1006525.s013.tif]
